# A Role of TGFß1 Dependent 14-3-3σ Phosphorylation at Ser69 and Ser74 in the Regulation of Gene Transcription, Stemness and Radioresistance

**DOI:** 10.1371/journal.pone.0065163

**Published:** 2013-05-31

**Authors:** Olena Zakharchenko, Monica Cojoc, Anna Dubrovska, Serhiy Souchelnytskyi

**Affiliations:** 1 Karolinska Biomics Center, Dept. of Oncology-Pathology, Karolinska Institute, Karolinska University Hospital, Solna, Stockholm, Sweden; 2 OncoRay-National Center for Radiation Research in Oncology, Medical Faculty Dresden Carl Gustav Carus, TU Dresden, Dresden, Germany; Shantou University Medical College, China

## Abstract

Transforming growth factor-β (TGFβ) is a potent regulator of tumorigenesis, although mechanisms defining its tumor suppressing and tumor promoting activities are not understood. Here we describe phosphoproteome profiling of TGFβ signaling in mammary epithelial cells, and show that 60 identified TGFβ-regulated phosphoproteins form a network with scale-free characteristics. The network highlighted interactions, which may distribute signaling inputs to regulation of cell proliferation, metabolism, differentiation and cell organization. In this report, we identified two novel and TGFβ-dependent phosphorylation sites of 14-3-3σ, i.e. Ser69 and Ser74. We observed that 14-3-3σ phosphorylation is a feed-forward mechanism in TGFβ/Smad3-dependent transcription. TGFβ-dependent 14-3-3σ phosphorylation may provide a scaffold for the formation of the protein complexes which include Smad3 and p53 at the Smad3-specific CAGA element. Furthermore, breast tumor xenograft studies in mice and radiobiological assays showed that phosphorylation of 14-3-3σ at Ser69 and Ser74 is involved in regulation of cancer progenitor population and radioresistance in breast cancer MCF7 cells. Our data suggest that TGFβ-dependent phosphorylation of 14-3-3σ orchestrates a functional interaction of TGFβ/Smad3 with p53, plays a role in the maintenance of cancer stem cells and could provide a new potential target for intervention in breast cancer.

## Introduction

TGFβ1 is a potent regulator of cell proliferation, death, migration and differentiation [1], [2]. TGFβ binds to serine/threonine kinase receptors on the cell surface. The complex of activated type I and type II TGFβ receptors phosphorylates a number of substrates and initiates intracellular signaling pathways, regulating transcription, protein synthesis, degradation and localization. The output of TGFβ1 treatment of cells is dependent on a type of cells and their status. The importance of Smad proteins has been shown, as well as a number of so-called Smad-independent pathways [2], [3]. In other words, the result of challenging of the cells with TGFβ1 depends on functional interactions between a number of components in cells, e.g. proteins.

Protein phosphorylation is one of the most crucial post-translational modifications in regulations of cellular functions. Phosphorylation at serine, threonine and tyrosine residues initiate conformational changes leading to changes in activity of proteins, and affect protein-protein and protein-nucleic acids interactions [4].

Proteomics has proven to be the only technology which is capable to provide a large-scale unbiased analysis of protein phosphorylation. Phosphopeptide- and phosphoprotein-based approaches have been employed with various degree of success [5], [6]. We reported previously modification of IMAC technique for enrichment of phosphorylated proteins [7] and the advantage of this phosphoprotein Fe-IMAC over a phosphopeptide studies is in providing information about full-length proteins and not selected sites/peptides. This is especially important for studies of proteins with many phosphorylation sites with different dynamics of phosphorylation, as each combination of phosphorylated sites will be well distinguishable for full-length proteins, but will be difficult to deduct from phosphopeptides.

Changing a cellular status, e.g. proliferation or inhibition of cell growth, requires coordinated changes of hundreds of proteins [8], [9]. Proteomics provides an overview of such alterations in protein expression and selected post-translational modifications. However, unveiling of key components in large datasets requires use of tools of systems biology. This includes various clustering methods, network building and modeling of relations [10], [11]. The principals underlining mechanisms of interaction between proteins have been extensively studied. The structure of protein-based networks is important for distribution of triggering signals to various cell function-controlling units, e.g. distribution of signals triggered by TGFβ1 to mechanisms regulating the cell cycle, differentiation, migration and apoptosis. Scale-free characteristics have been claimed for a number of networks, although scale-rich features have also been described [12]. Understanding of network features is of ultimate importance for unveiling of how an extracellular stimulus may trigger such different outputs, as inhibition of cell growth and stimulation of apoptosis.

Here we report a comprehensive phosphoproteomics screen of TGFβ1 signaling in MCF10A human breast epithelial cells. Systemic analysis showed that TGFβ1-regulated phosphoproteins form a scale-free network, which orchestrates cell metabolism, organization, development, proliferation, death and differentiation, response to stress, and various signaling pathways. The phosphoproteome analysis showed an importance of TGFβ1-dependent phosphorylation of 14-3-3σ for a signaling network, which contributes to regulation of gene transcription, tumorigenicity and DNA repair.

## Materials and Methods

### Cell cultures and antibodies

HEK293T, MCF7 and MCF10A cells were obtained from American Type Culture Collection (Manassas, VA). 293T and MCF7 cells were cultured in DMEM with 10% of fetal bovine serum, penicillin and streptomycin at concentration of 100 units/ml (Sigma-Aldrich). MCF10A cells were cultured in a MEGM medium (Lonza) supplemented with EGF, insulin, hydrocortisone, bovine pituitary extract and 5% horse serum. The antibodies used were: anti-Smad3 (#9513), anti-phospho-p53, Ser15 (#9286), anti-phospho-p53, Ser 392 (#9281) (Cell Signaling Technology/New England Biolabs); anti- 14-3-3σ (ab14123, Abcam); anti-c-Myc (C-19) (sc-788), anti- p-Tyr antibody (PY350) (sc-18182), anti-p-Thr antibody (H-2) (sc-5267), anti-p-Ser antibody (16B4) (sc-81514) (Santa Cruz Biotechnology), anti-flag (M5), anti-β-actin (Sigma-Aldrich), donkey anti-rabbit and sheep anti-mouse IgG, HRP-linked whole Ab (GE Healthcare).

### Luciferase reporter assay

Reporter assays with CAGA (12)-luc and E2F2-luc reporters were performed as described previously [36]. We used HEK293T cells, because they are responsive to TGFβ1 and are easily transfectable.

### Cell transfection

HEK293T cells were transfected in a 12 well plates using calcium phosphate–based transfection procedure. MCF10A cells were transfected in a 12 well plate by LipofectAMINE 2000 reagent. Medium was changed 6 hours after transfection and then cells were incubated in serum-free MEGM medium (Lonza) for 72 hours prior to addition of TGFβ1.

#### Cell proliferation assay

MCF10A proliferation in response to TGFβ1 treatment was measured by using CellTiter-Glo® Reagent (Promega) according to the manufacturer's recommendations. Cells were grown in DMEM/F12 medium supplemented with 15 mM HEPES buffer (pH 7.0), 10 ug/ml insulin, 20 ng/ml EGF and 0.5 µg/ml hydrocortisone, with and without TGFβ1 treatment at concentration of 5 ng/ml. Alternatively, proliferation of MCF10A cells in response to TGFβ1 treatment was analyzed by using Cell Titer 96® Non-Radioactive Cell Proliferation Assay (MTT) (Promega) according to the manufacturer's recommendations. MCF10A cells were cultured in a MEGM medium (Lonza) supplemented with EGF, insulin, hydrocortisone, bovine pituitary extract, and 5% horse serum, with and without TGFβ1 treatment at concentration of 5 ng/ml.

### GST-pull down assay

For GST-pull down assay GST, GST-Smad3, GST-Smad3MH1, GST-Smad3MH2 proteins were expressed in BL21(DE3) cells and purified according to standard protocols using Glutatione-Sepharose (GE Healthcare). HEK293T cells were transfected with pcDNA3.1 vector expressing 14-3-3σ-Flag protein. Total proteins from HEK293T cells were extracted using lysis buffer containing 1% NP-40, 50 mM Tris-HCl pH 8.0, 150 mM NaCl, 10 µg/ml aprotinin and 1 mM PMSF. The sepharose beads were added to the cell lysate (lysate from 6×10^6^ cells overexpressing 14-3-3σ protein per pull down) and incubated overnight at 4°C. After 3 washes with ice-cold lysis buffer, the samples were dissolved in a sample buffer for SDS-PAGE.

### Two-dimensional gel electrophoresis

Samples for two-dimensional gel electrophoresis were prepared according to the protocol described for Fe-IMAC [7]. Two-dimensional gel electrophoresis was performed as described earlier [7], [36]. Briefly, prepared samples were subjected to isoelectric focusing using IPGDry strips with immobilized pH gradient, pH range 3–10, 18 cm, linear (GE Healthcare). 2D-GE was performed according to the protocol described earlier [7], [36]. SDS–PAGE was performed in 12% polyacrylamide gels. After the electrophoresis, gels were fixed in 10% acetic acid and 20% methanol for 10–12 h. Proteins were detected by silver staining, as described earlier [7], [36]. Totally, 6 gels with samples from three experiments were prepared and subjected to analysis.

### Gel analysis

Silver stained gels were scanned and analyzed by the ImageMaster 2D Platinum Version 6.0 (GE Healthcare). Gels that did not show deviations in pattern of protein migration were used to generate master gels of the phosphoproteome of cells treated or not treated with TGFβ1. Proteins changing their phosphorylation after treatment with TGFβ1 were considered for identification. Statistical significance of changes was evaluated using the ImageMaster 2D Platinum Version 6.0 software.

### Protein identification

Protein spots were excised from the gels, destained and subjected to in-gel digestion with trypsin (modified, sequence grade porcine, Promega, USA), as described earlier [7], [36]. Tryptic peptides were concentrated and desalted on a “nano-column”, i.e. ZipTip. Peptides were eluted with 65% acetonitrile, containing the matrix a-cyano-4-hydroxycinnamic acid, and applied directly onto the metal target and analyzed by MALDI TOF MS on a BrukerBiflex (BrukerDaltonics). Peptide spectra were internally calibrated using autolytic peptides from the trypsin. The proteins were identified in NCBInr sequence database using ProFound (http://65.219.84.5/service/prowl/profound.html). One miscut, alkylation, and partial oxidation of methionine were allowed. Significance of the identification was evaluated according to the probability value, “Z” value, and sequence coverage.

### Pathway analysis

Functional and pathway analysis was performed using Ingenuity Pathway Analysis (www.ingenuity.com). A data set containing identified proteins was uploaded into the Ingenuity Pathway Analysis application and TGFβ1-dependent networks regulating cell proliferation, death, migration and differentiation were generated. Fischer's exact test was used to calculate a p-value determining the network connectivity.

### Two-dimensional Phosphopeptide Mapping

Metabolic labeling of cells with [^32^P] orthophosphate (GE Healthcare) was performed as described previously [36]. Briefly, radioactively labeled 14-3-3σ proteins were subjected to digestion with trypsin (modified, sequence grade porcine; Promega), and the tryptic digest was separated on thin-layer cellulose plates by electrophoresis and chromatography. Plates were exposed in a FujiX2000 PhosphoImager (Fuji). Phosphopeptides of interest were subjected to phosphoamino acid analysis and to Edman degradation.

### In vivo tumorigenicity experiments

MCF7 cells were stably transduced with retrovirus expressing 14-3-3σ WT, 14-3-3σ S69/74A, 14-3-3σ S69A, 14-3-3σ S74A or with GFP expressing vector as a control. Stable high GFP-expressing MCF7 cells were collected by FACS sorting. The estrogen pellets (17ß-estradiol, 0.1 mg per pellet, 21 d release; Innovative Research of America, Sarasota, FL) were implanted at the neck of 5–8 weeks old NOD.CB17-Prkdc (SCID) mice under anesthesia. For subcutaneous (s.c.) tumor development, 10^6^ MCF7 cells expressing 14-3-3σ WT, 14-3-3σ S 69/74 A, 14-3-3σ S69A, 14-3-3σ S74A or GFP were embedded into 100 µl of BD Matrigel and injected s.c. Each experimental group contained at least five mice. Animal studies were performed in accordance with the animal protocols (P06-147 and P09-243) approved by the GNF (Genomics Institute of the Novartis Research Foundation, San Diego, California, United States of America) Institutional Animal Care and Use Committee for this study.

### Sphere formation assay

Single cell suspension was plated at 500 cells/2 mL per well in triplicates in 6 well low-attachment plates (Fisher Scientific). Cells were grown in serum-free Mammary Epithelial Basal Medium (MEBM, Lonza) supplemented with 4 µg/mL insulin (Sigma), B27, 20 ng/mL EGF, 20 ng/mL FGF (Life Technologies). Spheres were analyzed after 14 days.

### Colony formation assay

Cells were plated in 6 well plates at 500 cells per well in triplicate and grown in DMEM medium containing 10% FBS for 14 days. The cell were fixed with 10% formalin for 30 min and stained with 0.05% crystal violet for 30 min, then washed twice with distilled water and air dried.

### Flow cytometry analysis of ALDH activity

For flow cytometry, cells were dissociated with trypsin and washed 2 times in solution containing Ca^2+^ and Mg^2+^-free PBS with 1 mM EDTA, 25 mM HEPES buffer (pH 7.0) (Gibco BRL), and 1% FBS. Cells were stained live at the concentration of 1×10^6^ cells/ml in the Aldefluor assay buffer according to the protocol recommended by the manufacturer. Samples were analyzed on a BD LSR II flow cytometer. Aldefluor staining was detected in GFP fluorescence channel, and the samples treated with the inhibitor DEAB were used as controls to set the gates defining the ALDH^+^ region. FlowJo 7.2.5 software was used to analyze the data.

### Histology

For cryosectioning, the tumors were fixed by immersion in 4% paraformaldehyde, cryoprotected in 20% sucrose, frozen and embedded in sucrose: OCT (1∶1). Cryostat sections (12 µm) were collected on Superfrost plus slides. Slides were blocked for 30 min in antibody buffer (50 mM NaCl, 50 mM Tris Base, 1% BSA, 100 mM L-Lysine, 0.04% sodium azide [pH 7.4]) containing 0.4% TritonX100 and 10% horse serum and then incubated overnight at 4°C with the primary antibodies anti-CD24 (555426, BD Bioscience, dilution 1∶200) and anti-CD44 (ab24504, Abcam, dilution 1∶100). Slides were washed and inclubated with secondary antibodies conjugated with Alexa 488 or 555 (1∶1000; Molecular Probes) diluted in antibody buffer at room temperature for 1 h. TUNEL assay was performed using The DeadEnd Fluorometric TUNEL System (Promega) according to manufacturer's recommendations. Tissue staining was analyzed using an UltraVIEW VoX confocal microscope (PerkinElmer). For quantification, cells in at least four randomly selected fields of view were counted for each condition. At least 500 cells per condition were counted.

### Immunofluorescent microscopy

MCF7 cells were plated in a 96 well black clear bottom plate (Greiner Bio-One) at a density of 1000 cells/well in medium containing 10% serum. The cells were fixed for 30 min in 3.7% formaldehyde at room temperature and permeabilized with 0.125% Triton X-100 for 10 min. The cells were washed with PBS and blocked with 10% BSA in PBS for 1 h. The cells were then incubated with primary antibody diluted in 3% BSA in PBS (anti-Smad3, New England Biolabs, dilution 1∶100) and anti- phospho-histon H2AXS139 (Upstate, Hamburg, Germany, dilution 1∶100) overnight at 4°C and washed ten times with PBS. Cells were then incubated for 1 h with a secondary antibody conjugated with Alexa 488 or 555 (Invitrogen) diluted 1∶500 in 3% BSA in PBS. After extensive washes with PBS the cells were stained with DAPI and examined under epifluorescent illumination. For quantification, at least 100 cells per condition were counted.

### Cell irradiation

Irradiation was performed at room temperature, using single doses of 200-kV X-rays (Yxlon Y.TU 320; Yxlon) filtered with 0.5 mm copper. The dose-rate was approximately 1.3 Gy/min at 20 mA and applied dose was 4 Gy.

### Immunobloting and immunoprecipitation

For immunoblotting, cell lysates were resolved on SDS polyacrylamide gels and transferred onto Hybond P membranes (Amersham Biosciences, Piscataway, NJ). Membranes were blocked with 5% (w/v) BSA for one hour and then incubated with primary antibody against target proteins with dilution as recommended by manufacturer followed by an HRP-conjugated secondary antibody (GE Healthcare, Uppsala, Sweden). The proteins were visualized using Western Blotting Luminol Reagents (Santa Cruz Biotechnology Inc.). For immunoprecipitation, cell lysates were incubated with antibodies against target proteins and protein A-Sepharose beads (Sigma-Aldrich) for 6 hours at 4°C with end-over-end rotation. Immunocomplexes bound to protein A-Sepharose beads were collected by centrifugation and washed 3 times in lysis buffer before being resolved by SDS-PAGE.

### Blue native polyacrylamide gel electrophoresis

MCF10A cells were treated with TGFβ at concentration 5 ng/ml for 24 hours. Cells were subsequently rinsed twice with ice-cold PBS and incubated with solubilization buffer A (50 mM NaCl, 2 mM 6-aminohexanoic acid, 1 mM EDTA, 50 mM imidazole/HCl, pH 7.0) for 15 min on ice. Cells lysates were clarified by centrifugation and incubated with Triton X-100 at a final concentration 3% for 15 min on ice for solubilization of protein complexes. Glycerol at a final concentration 5.4% and Coomassie brilliant blue at a final concentration 0.25% were added to the sample prior to loading. The protein complexes were separated by SDS-PAGE using 3.5%–13% linear gradient gels. Electrophoresis was performed using anode buffer (25 mM imidazole/HCl, pH 7.0) and cathode buffer B (50 mM tricine, 7.5 mM imidazole, 0.02% Coomassie brilliant blue dye, pH 7.0). When Coomassie dye migrated about one third of the gel length, cathode buffer B was replaced with cathode buffer B/10 (50 mM tricine, 7.5 mM imidazole, 0.002% Coomassie brilliant blue dye, pH 7.0).

### RT-PCR

Total RNA was prepared using the QiagenRNeasy Mini kit. RT-PCR was performed using SYBRGreen I Dye (Applied Biosystems) according to the manufacturer's instructions. The following primers were used for analysis of gene expression: 5′- TTACGCCGCTGACATTGTGTT-3′ (COL7A1 forward), 5′- ACCAGCCCTTCGAGAAAGC-3′ (COL7A1 reverse); 5′- CATCCCCCATCCTACGTGG-3′ (PAI-1 forward), 5′- CCCCATAGGGTGAGAAAACCA-3′ (PAI-1 reverse); 5′- CCTGTCACTGTCTTGTACCCT-3′ (p21 forward), 5′- GCGTTTGGAGTGGTAGAAATCT -3′ (p21 reverse).

### Statistical analysis

The results of luciferase activity assay, cell proliferation assay, flow cytometry analysis, colony formation assay, sphere formation assay, immunofluorescent microscopy and histological analysis and *in vivo* tumorigenicity assays were analyzed by paired t-test. A p-value<0.05 was regarded as statistically significant.

## Results

### Phosphoproteome profiling of TGFβ1 signaling

**Figure 1:**
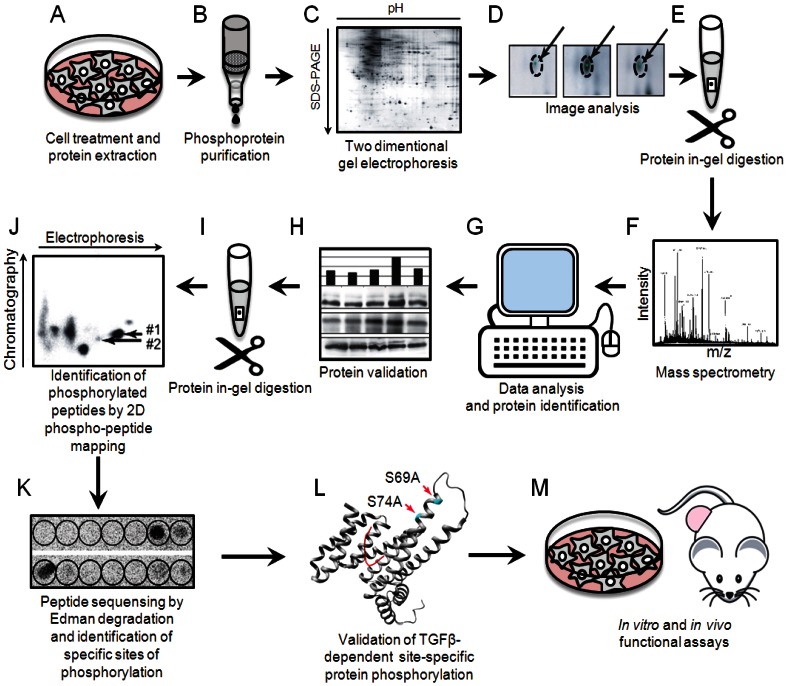
Outline of the experimental workflow for phosphoproteome profiling of TGFβ1 signaling.

We generated phosphoprotein expression maps using immobilized metal-affinity chromatography technique developed by our group [7]. Outline of the experimental workflow for phosphoproteome profiling of TGFβ1 signaling is shown in Figure 1.

MCF10A cells were treated with TGFβ1 for 15, 30, 60 and 120 minutes and phosphorylated proteins were enriched by Fe-IMAC (Figure S1, S2). We detected in average 393 phosphoprotein spots in non-treated cells, 370 after 15 min, 371 after 30 min, 474 after 60 min and 436 after 120 min of treatment of cells. Although most of the protein spots migrated in the region of 2D gels corresponding to pI lower than 7.0, this shift was not dominant. The presence of the proteins of various molecular masses in 2D gels indicated that upon Fe-IMAC enrichment was no selection related to the size of proteins. An increase in the number of phosphorylated proteins upon TGFβ1 treatment was expected, as TGFβ1 activates serine/threonine kinase receptors. However, a slight decrease in the number of phosphoproteins during the first 30 min indicated that de-phosphorylation of proteins had occurred. This is an important observation, as it showed that TGFβ1 initiated both phosphorylation and de-phosphorylation events, in contrary to the previous suggestion of predominantly phosphorylation-inducing signaling [1], [2], [3].

We confirmed that Fe-IMAC-enriched proteins are phosphoproteins by performing [^32^P] orthophosphate labeling followed by Fe-IMAC and 2D gel electrophoresis. After exposure in a phosphorimager, co-migration of silver-stained and ^32^P-labeled spots indicated that Fe-IMAC-enriched proteins were phosphorylated. Our control mass spectrometry analysis of protein phosphorylation showed significant variability in phosphopeptide detection. This was expected due to the well known phenomenon of variability in peptide ionization. The mass spectrometry-based phosphopeptide analysis was found inferior to the phosphoprotein approach in detection and coverage of phosphorylated proteins (data not shown). Thus, two different phosphorylation-specific techniques provide confirmation of phosphorylation of detected proteins, i.e. directed detection of presence of phosphoryl groups (^32^P) in proteins and previously confirmed high specificity of used by us Fe-IMAC protocol [7].

Gel image analysis identified 85 protein spots which changed their appearance upon treatment of cells with TGFβ1. The spots were selected for analysis of proteins if TGFβ1 induced changes of more than 50% of their level of phosphorylation in at least one of the time-points of treatment, as compared to any of the other time-points. The level of phosphorylation was defined as a volume of a protein spot in a Fe-IMAC 2D gel. For identification of proteins we used peptide mass fingerprinting by MALDI TOF mass spectrometry, and each protein was identified in at least two different preparations of respective phosphoprotein spots. Thereby, we identified 60 unique proteins in 85 protein spots (Table 1).

**Table 1:** Proteins identified as changing their phosphorylation upon TGFβ1 treatment.

No a)	Protein Identity b)	Accesion No. b)	Probability b)	Est'd Z b)	Sequence coverage, %	Theoretical value		Experimental value		Matched peptides
						pI	M_r_(kDa)	pI	M_r_(kDa)	
1	Heat shock 70 kDa protein 8 isoform 2	NP_69488.1	1.0e+000	2.16	36	5.6	53.61	4.8	60	15
2	Heat shock 70 kDa protein 9B (mortalin-2)	AAH30634.1	1.0e+000	2.28	34	6.0	74.12	5.0	60	21
3	Mitosis-specific chromosome segregation protein SMC1 homolog	AAB34405.1	1.0e+000	2.28	18	7.6	143.81	6.5	47.5	17
4	Keratin 14	AAP35850.1	1.0e+000	2.39	38	5.1	51.92	4.8	47	27
5	ENO-1	AAH50642.1	1.0e+000	1.98	35	7.0	47.49	7.5	45	13
6	ENO-1	AAH50642.1	1.0e+000	2.15	42	7.0	47.49	7.4	45	18
7	Eukaryotic translation initiation factor 3, subunit 5 epsilon (EIF3S5)	AAP35540.1	1.0e+000	1.97	27	5.2	37.13	4.7	37	10
8	ENO-1	AAH50642.1	1.0e+000	2.08	49	7.0	47.49	7.3	45	19
9	Keratin 5	AAH71906.1	9.6e−001	1.45	24	7.7	62.59	7.8	40	15
10	A+U-rich element RNA binding factor	BAA22860.1	1.0e+000	2.43	29	8.9	30.34	7.0	32	10
11	Annexin A2, isoform 2	AAH23990.1	1.0e+000	2.30	35	7.7	38.79	7.2	32	15
12	CNN2 (calponin 2)	CAG46609.1	1.0e+000	1.04	24	7.0	34.09	7.0	32	10
13	Keratin 10	AAA59199.1	1.0e+000	1.70	18	4.7	39.84	8.0	26	9
14	Peroxiredoxin 6	NP_004896.1	1.0e+000	1.44	37	6.0	25.13	6.5	25	8
15	Peroxiredoxin 1	CAI13096.1	1.0e+000	2.26	44	6.4	19.13	9.0	19	10
16	Keratin 14	NP_000517.2	1.0e+000	2.35	40	5.1	51.89	4.6	45	26
17	Keratin 17	NP_000413.1	1.0e+000	2.32	39	5.0	49.37	4.3	40	23
18	Laminin-binding protein	CAA43469.1	9.7e−001	0.69	20	4.8	31.89	4.0	33	6
19	RAB6-interacting protein 2 (RAB6IP2)	AAH68006.1	9.4e−001	0.61	19	6.8	71.88	4.8	33	14
20	Eukaryotic translation initiation factor 3, subunit 2 beta (EIF3S2)	NP_003748.1	1.0e+000	2.22	25	5.4	36.88	5.2	33	14
21	Radical S-adenosyl methionine and flavodoxin domains 1 (RSAFD1)	AAH51888.1	9.9e−001	0.70	17	5.8	44.08	5.0	32	8
22	Eukaryotic translation initiation factor 3, subunit 2 beta (EIF3S2)	NP_003748.1	1.0e+000	1.68	39	5.4	36.88	4.2	32	13
23	Keratin 10, typeI, cytoskeletal	KRHU0	1.0e+000	1.87	26	5.2	59.74	5.2	26	17
24	Zinc finger protein 62 homolog	NP_689496.1	1.0e+000	0.86	16	9.9	58.78	8.3	20	9
25	RAB6-interacting protein 2 isoform alpha	NP_055879.1	6.3e−001	0.74	15	6.2	108.89	8.3	20	14
26	Antigen MLAA-34	AAQ93064.1	9.0e−001	0.93	26	8.7	39.24	8.0	18	8
27	Eukaryotic translation initiation factor 4H, isoform 2	AAH10021.1	1.0e+000	2.19	44	7.8	25.24	8.0	25	9
28	Keratin 10, type I, cytoskeletal	KRHU0	1.0e+000	1.78	22	5.2	59.74	9.5	20	12
29	Heterogeneous nuclear ribonucleoprotein A2/B1 isoform A2	NP_002128.1	1.0e+000	1.84	20	8.7	36.05	9.2	30	8
30	Heterogeneous nuclear ribonucleoprotein A2/B1 isoform A2	NP_002128.1	1.0e+000	2.36	48	8.7	36.05	9.3	30	13
31	Heterogeneous nuclear ribonucleoprotein A2/B1 isoform A2	NP_002128.1	1.0e+000	2.18	40	8.7	36.05	9.0	32	15
32	Heterogeneous nuclear ribonucleoprotein A2/B1 isoform A2	NP_002128.1	1.0e+000	2.31	29	8.7	36.05	9.2	29	11
33	Heterogeneous nuclear ribonucleoprotein A2/B1 isoform A2	NP_002128.1	1.0e+000	2.38	42	8.7	36.05	8.7	28	12
34	Heterogeneous nuclear ribonucleoprotein A2/B1 isoform A2	NP_002128.1	1.0e+000	1.09	15	8.7	36.05	8.7	25	7
35	Human Muscle Fructose 1.6-Biphosphate Aldolase Complexed with Fructose 1,6-Biphosphate	4ALD	1.0e+000	2.09	28	8.8	39.73	8.8	35	10
36	Human Muscle Fructose 1.6-Biphosphate Aldolase Complexed with Fructose 1,6-Biphosphate	4ALD	1.0e+000	2.25	24	8.8	39.73	9.1	35	9
37	Human Muscle Fructose 1.6-Biphosphate Aldolase Complexed with Fructose 1,6-Biphosphate	4ALD	1.0e+000	1.28	22	8.8	39.73	9.0	35	8
38	Vasodilator-stimulated phosphoprotein	CCA67147.2	1.0e+000	2.15	26	9.2	39.76	9.5	45	6
39	Vasodilator-stimulated phosphoprotein	CCA67147.2	1.0e+000	1.77	21	9.2	39.76	9.3	45	11
40	Keratin 10, type I, cytoskeletal	KRHU0	8.6e−001	0.60	13	5.2	59.74	7.2	46	8
41	ENO 1	AAH50642.1	1.0e+000	2.35	43	7.0	47.49	6.8	38	19
42	ENO 1	AAH50642.1	1.0e+000	2.40	35	7.0	47.49	7.0	38	14
43	Inosine monophosphate (IMP) dehydrogenase 2	AAH155567.1	9.7e−001	0.69	12	6.4	56.24	6.5	47	8
44	DEAD (Asp-Glu-Ala-Asp) box polypeptide 48	NP_055555.1	9.9e−001	0.79	20	6.3	47.25	6.3	47	7
45	Stress-induced phosphoprotein 1 (Hsp70/Hsp90-organining protein)	AAV38814.1	1.0e+000	1.98	31	6.4	63.25	6.5	50	20
46	Stress-induced phosphoprotein 1 (Hsp70/Hsp90-organining protein)	AAV38814.1	1.0e+000	1.98	33	6.4	63.25	6.7	50	20
47	Fumarate hydratase	AAP88841.1	1.0e+000	1.32	17	9.0	54.79	7.5	40	9
48	Kerain 10, type I, cytoskeletal 10	K1CJ	1.0e+000	1.14	13	5.1	59.73	7.2	40	9
49	Zinc finger protein 62 homolog	NP_689496.1	9.7e−001	0.63	19	9.9	58.78	7.8	35	9
50	Antigen MLAA-34	AAQ93064.1	9.7e−001	0.61	18	8.7	39.24	6.8	30	6
51	Cytokeratin 9	AAC60619.1	1.0e+000	1.83	23	5.1	62.20	6.5	33	8
52	RNA binding protein 4	AAH32735.1	1.0e+000	1.94	23	6.6	40.70	6.6	33	9
53	Keratin 9	NP_000217.2	1.0e+000	2.18	26	5.1	62.27	6.9	33	14
54	Qntigen MLAA-34	AAQ93064.1	9.8e−001	0.68	23	8.7	39.24	7.3	27	6
55	Zinc finger protein 62 homolog	NP_689496.1	1.0e+000	0.99	18	9.9	58.78	7.2	26	8
56	Guanyl nucleotide releasing protein 4	AAL87858.1	9.4e−001	0.51	9	9.1	76.22	7.5	27	9
57	Transcription factor NRF	AAH47878.1	9.6e−001	0.57	11	9.2	78.34	7.9	28	7
58	Heterogeneous nuclear ribonucleoprotein A2/B1 isoform A2	NP_002128.1	1.0e+000	2.31	34	8.7	36.05	7.9	30	11
59	Heat shock 70 kDa protein 9B (mortalin-2)	AAH30634.1	1.0e+000	2.26	41	6.0	74.12	5.0	48	25
60	Heat shock 70 kDa protein 9B (mortalin-2)	AAH30634.1	1.0e+000	2.26	33	6.0	74.12	4.5	60	19
61	Heat shock 70 kDa protein 9B precursor (MTHSP75)	AAA67526.1	1.0e+000	2.37	18	6.0	74.05	5.0	50	14
62	Heat shock 70 kDa protein 9B (mortalin-2)	AAH30634.1	1.0e+000	1.71	17	6.0	74.12	5.3	50	11
63	Unknown (chaperonin containing TCP1)	AAH02971.1	1.0e+000	1.99	23	5.4	59.91	5.2	47	12
64	Prolyl 4-hydroxylase, beta subunit	NP_000909.2	1.0e+000	2.28	32	4.8	57.50	4.0	47	17
65	Chaperonin containing TCP1, subunit 2 (beta)	AAI13517.1	1.0e+000	0.86	16	9.9	58.78	8.3	20	10
66	Tubulin, beta polypeptide	NP_821133.1	1.0e+000	2.39	38	4.8	50.11	4.3	46	34
67	Keratin 7	NP_005547.2	1.0e+000	2.39	35	5.4	51.46	5.0	46	17
68	Heterogeneous nuclear ribonucleoprotein K isoform a	NP_112553.1	1.0e+000	2.32	28	5.2	51.30	7.5	46	13
69	Gamma-actin	JC5818	1.0e+000	1.80	35	5.3	41.99	5.0	35	10
70	Ribosomal protein P0	AAH01127.1	1.0e+000	1.98	34	5.4	34.43	5.3	31	10
71	Ribosomal protein P0	AAH01127.1	1.0e+000	1.98	34	5.4	34.43	5.5	31	9
72	SFN (14-3-3 sigma, stratifin)	AAH01550.1	1.0e+000	2.18	37	4.8	24.38	6.5	32	10
73	Eukaryotic translation elongation factor 1 beta 2	AAP35742.1	1.0e+000	1.54	29	4.5	24.92	3.7	25	8
74	SFN (14-3-3 sigma, stratifin)	AAH01550.1	1.0e+000	2.18	45	4.8	24.38	4.2	24	21
75	Mitochondrial short-chain enoyl-coenzyme A hydratase 1 precursor	NP_004083.2	1.0e+000	2.29	50	8.9	31.81	6.0	24	16
76	Proteasome alpha 5 subunit	NP_002781.2	1.0e+000	2.29	50	8.9	31.81	6.0	24	16
77	Ribosomal protein S6 kinase, 90 kda, polypeptide 3	BAC81131.1	1.0e+000	1.05	16	6.4	82.48	6.3	10	10
78	Keratin9	NP_00217.2	1.0e+000	1.14	18	5.1	62.27	9.0	10	14
79	Chain B Human Mitochondrial Single Strand DNA Binding Protein (HMSSB)	1S3O_B	1.0e+000	1.98	49	8.2	15.18	9.2	8	7
80	RAS guanyl releasing protein 4	AAK85701.1	9.7e−001	0.63	11	9.0	11.79	9.0	7	10
81	Fk506 Binding Protein Fkbp Mutant R42kH87V Complex With Immunosuppressant Fk506	1BKF_A	1.0e+000	2.24	46	8.1	11.79	9.0	7	10
82	Heat shock 70 kDa protein 8 isoform 1	NP_006588.1	1.0e+000	2.37	32	5.4	71.11	4.1	63	19
83	Eukaryotic translation elongation factor 2	NP_001952.1	8.2e−001	0.64	13	6.4	96.29	3.9	25	11
84	Endoplasmic reticulum protein 29 precursor	NP_006808.1	1.0e+000	2.26	34	6.8	29.03	6.2	25	10
85	Lamin A/C isoform 2	NP_005563.1	1.0e+000	2.31	35	6.4	65.17	6.6	49	18

a )- selected phosphoprotein spots from 2-D gels;

b )-NCBI sequence identification numbers.

Probability, Z-value, coverage and theoretical pI and M_r_ were obtained from ProFound search. The calculation of experimental pI and M_r_ was based on migration of proteins on 2-D gels.

Thirteen proteins were identified in multiple spots, with heterogenous nuclear ribonucleoprotein A2/B1 identified in 7 protein spots, enolase-1 in 5 spots, HSP-70 in 4 spots, MLAA-34 antigen and fructose 1,6-biphosphate aldolase in 3 spots each, eukaryotic translation initiation factor 3, keratin 10, keratin 9, zink finger protein 62, vasodilator-stimulated phosphoprotein, stress-induced phosphoprotein 1, ribosomal protein P0 and 14-3-3σ in 2 spots each (Table 1). An identification of the same protein in different spots was a strong indication of phosphorylation at multiple sites and may indicate combinations of phosphorylated sites.

Phosphorylation may affect apparent molecular mass of a protein upon migration in SDS-PAGE, which may result in deviation of observed molecular mass from theoretical one. We observed such deviations for a number of identified proteins (Table 1). However, we also observed that TGFβ1 affected appearance of phosphorylated fragments of proteins, e.g. HSP-70 and cytokeratin 9. This corroborates importance of studying of the full-length proteins, as performed in this work. Phosphorylation of selected identified proteins was validated by immunobloting of MCF10A cell extracts with anti-phosphoSer/phosphoThr/phosphoTyr antibodies (FKBP12, Actin, Enolase1, 14-3-3σ; Figure S3). Thus, we identified 60 unique proteins, which phosphorylation is regulated by TGFβ1.

### Systemic analysis of TGFβ1 targets

TGFβ affects practically all cellular functions, often having both stimulatory and inhibitory effects, e.g. proliferation, apoptosis, differentiation and migration [1], [2], [3]. To gain insights into the mechanisms of TGFβ action, we performed a systemic analysis of our phosphoproteomics data. This included functional and dynamics clustering, building of a network of relationship between identified TGFβ1-regulated proteins, and analysis of systemic properties of the network.

Functional clustering showed that TGFβ1 affected phosphorylation of proteins involved in primary cellular metabolic processes, cell organization, development, differentiation, signal transduction, cell proliferation, cell cycle, cell death, transport and motility (Figure S4A). Dynamics of protein phosphorylations were variable, without predominant up- or down-regulation (Figure S4B, C). Dynamics of protein phosphorylation in selected functional clusters was also variable; as an example, dynamics of cell proliferation- or apoptosis-regulating proteins is shown (Figure S4B, C). It has to be noted that the most of the identified proteins and their phosphorylation have not been earlier described as components of TGFβ1 signaling, which makes predictions of functional input of this phosphorylation uncertain and requires separate detailed study of each protein. However, our description of the TGFβ1-regulated phosphoproteins is the first step in building a comprehensive regulatory network dependent on phosphorylation. Our observation showed also that TGFβ1-dependent phosphorylation had a similar high dynamics of phosphorylation reported for other regulatory systems, e.g. EGF signaling [13], [14].

Large-scale analysis of identified phosphoproteins showed that they form a network with scale-free characteristics (Figure S5). The network consists of 102 species (proteins or their genes), with 58 species identified as functional or physical interactors with TGFβ1-regulated proteins, e.g. “guilt by association”, in addition to identified by us proteins (Table 1). Two clusters including elongation initiation factors and chaperons were detected (Figure S5). The average number of connections (or strings) for a single species in the whole network is 9 and for the identified proteins the average number of connections is 3. This indicates that by generation of the network we detected highly connected hubs which otherwise would not be identified. The average number of intermediate connections between two TGFβ1-regulated proteins is 2.4, suggesting that all TGFβ-dependent phosphoprotein-inputs are closely connected.

**Figure 2:**
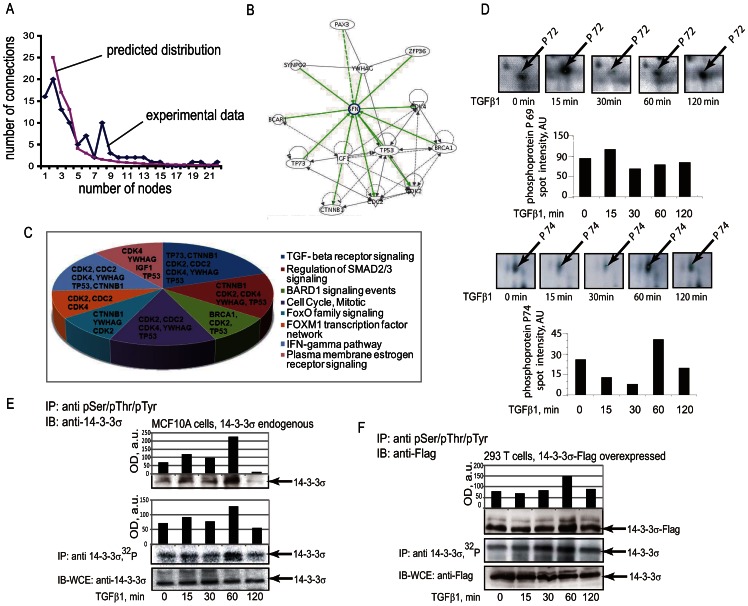
14-3-3σ sub-network of TGFβ1-regulated phosphoproteins. (**A**) Graphs show distribution of connections for species of the network. Distribution for proteins identified by phosphoproteomics (rhombs; experimental data) and as would be expected by a power law distribution of connections of species in an ideal scale-free network (squares; predicted distribution) are shown. (**B**) A sub-network of 14-3-3σ (SFN). Proteins which are in proximal dependencies to 14-3-3σ were extracted from the complete network (Figure S5) into the presented sub-network. (**C**) Signaling pathways involving the proteins assembled in the 14-3-3σ sub-network. The analysis was performed using Gene Set Analysis Toolkit V2 software. (**D**) Validation of 14-3-3σ phosphorylation upon treatment of cells with TGFβ1. Images of the areas of 2D Fe-IMAC gels with annotation of phosphoprotein spots p72 and p74, in which 14-3-3σ was identified are shown. Values of the protein spot volumes are shown below images of gels for both protein spots. (**E**) Phosphorylation of endogenous 14-3-3σ in MCF10A cells was evaluated by immunoprecipitation with anti-phosphoserine, threonine and tyrosine antibodies (upper panel) or by incorporation of ^32^P (middle panel). Control immunoprecipitation of 14-3-3σ is shown in lower panel. Densitometry analysis of the protein immunoblots or ^32^P incorporation is shown in accompanying graphs. (**F**) Phosphorylation of Flag-14-3-3σ fusion protein expressed in HEK293T cells was evaluated in the same way as for endogenous protein. The upper panel shows detection of phosphorylation by immunoprecipitation and the middle panel shows incorporation of ^32^P. The lower panel shows expression of 14-3-3σ. Migration positions of 14-3-3σ are shown by the arrows and treatments with TGFβ1 are indicated. Densitometry analysis of the protein immunoblots is shown in accompanying graphs. Representative experiments out of 3 performed are shown.

Distribution of node connections showed that the network contains highly connected hubs which appeared in the network with a frequency higher than it would be expected by a power law relationship (Figure 2A). The network analysis pointed to TGFβ as one of the main hubs, although TGFβ ligand itself was not in the experimental dataset. This strongly indicated that we detected functional dependencies previously assigned to TGFβ and provided confidence that we were able to identify previously reported TGFβ-specific activities.

Among other highly connected growth factor species in the network were found EGF, TNF, IGF receptor and IL8. This suggests that these growth factors and TGFβ may converge on the same components of the network. For example, RPSA, RPS6KA3, BRCA1, CDK2,RET and HNRPK are novel predicted convergence points for TGFβ and EGF, in addition to those species which have been described earlier, e.g. H-Ras, AKT1, Src and NF-kB. For TNF and TGFβ signaling predicted convergence points are CDKN2A, P4HB, SMC1B, 14-3-3σ, PRDX6, CAST, RPSA, PCDS, CREBP, Src, hnRNPK, NFkB and CLEC11A. For IGF and TGFβ signaling they are ANXA2, NDY, Src, ALDOA and CCNA1. And for IL8 and TGFβ, they are TPT1, NOS2A, NKRF, RLDZ, NFB, AKT1 and Src.

Thus, systemic network analysis predicted that TGFβ1-dependent phosphorylation might affect in a coordinated way the various cellular processes. Our results showed also that the TGFβ1 initiated a network signaling with predominantly scale-free characteristics. The proteins with higher than expected connectivity represent potential key hubs of the network, so-called “drivers”. Our results pointed also to intersection components between TGFβ and EGF, TNF, IGF and IL8 signaling.

### TGFβ1 induced phosphorylation of 14-3-3σ at Ser69 and Ser74

Network analysis indicated a potential role of 14-3-3σ in regulation of cell proliferation with involvement of p53 (Figure 2B). We selected 14-3-3σ for further analysis, as it has been most directly linked to cancer of all the 14-3-3 genes. The high frequency of 14-3-3σ inactivation by epigenetic silencing or p53 mutations indicates that it has a critical role in tumor formation [15], [16]. Analysis of the signaling pathways of the 14-3-3σ sub-network provided a link between the control of the cell cycle, TGFβ signaling and DNA repair mechanisms (Figure 2C). In addition, the 14-3-3σ sub-network was predicted to regulate FOXO signaling and FOXM1 transcription factor networks that are known marker regulators of cancer progenitor populations and metastasis (Figure 2C).

First, we confirmed TGFβ1-dependent phosphorylation of endogenous 14-3-3σ protein in MCF10A cells. In 2D gels, 14-3-3σ was identified in two protein spots. Notably, p72 migrated at a position corresponding to molecular mass of 32 kDa and pI 6.5, while p74 spot migrated at 24 kDa and pI 4.2 position (Figure 2D). These two forms of 14-3-3σ are believed to be due to post-translational modifications, e.g. phosphorylation. In 1D SDS-PAGE, these two forms were detected as a single band, and phosphorylation status is a sum of these two forms. Difference in the migration positions may be explained by differences in the patterns of post-translational modifications and solubilization efficacy of 14-3-3σ in urea and in SDS-containing solvents. We observed increased phosphorylation of 14-3-3σ after TGFβ1 treatment for 1 h using two types of assays. First, the phosphorylated proteins were immunoprecipitated with anti-phosphoserine, anti-phosphothreonine and anti-phosphotyrosine antibodies, followed by immunoblotting with antibodies to endogenous 14-3-3σ (Figure 2E). In the second assay, cells were metabolically labeled with [^32^P] orthophosphate, 14-3-3σ was precipitated with specific antibodies and detected after exposure in a phosphorimager (Figure 2E). Similar TGFβ1-dependent induction of 14-3-3σ phosphorylation was observed for an ectopically expressed 14-3-3σ (Figure 2F); ectopically expressed Flag-14-3-3σ was in excess as compared to the endogenous 14-3-3σ (data not shown). Phosphorylation of transfected 14-3-3σ was evaluated in assays of the same types as assays of phosphorylation of endogenous protein. Thus, the phosphorylation pattern of 14-3-3σ was confirmed for both endogenous and ectopically expressed protein.

**Figure 3:**
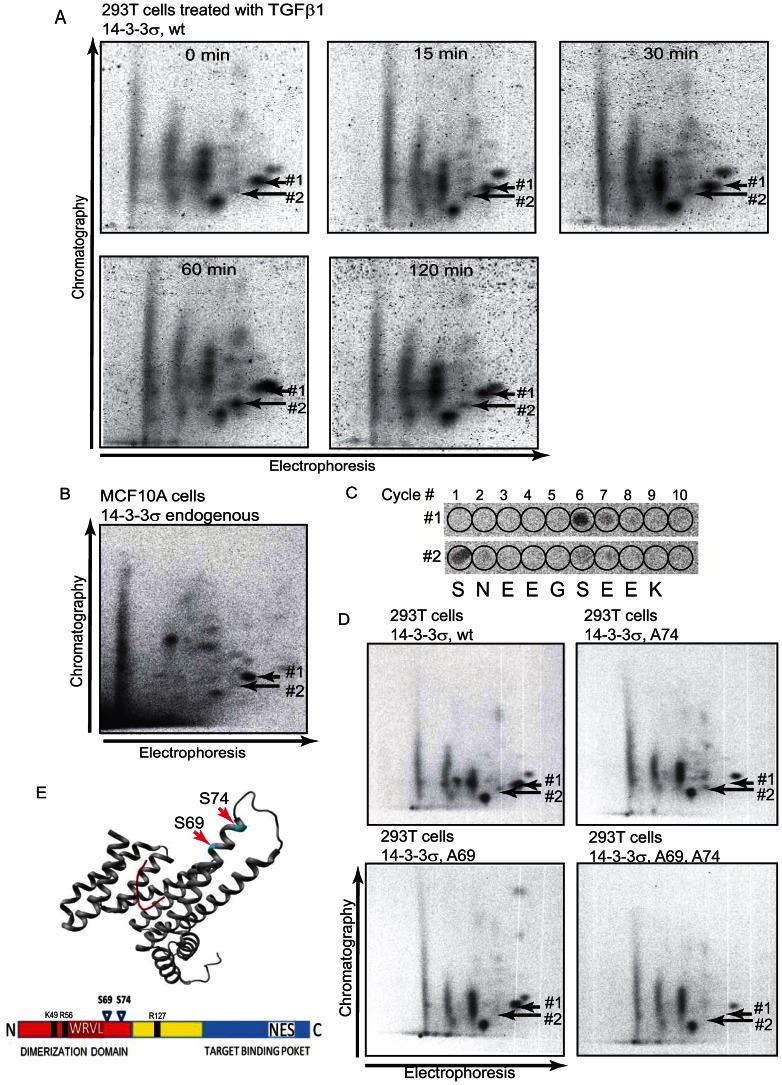
14-3-3σ is phosphorylated at Ser69 and Ser74. (**A**) Two-dimensional phosphopeptides mapping showed appearance of 2 phosphopeptides upon TGFβ1 treatment. Migration positions of these phosphopeptides are shown by arrows, as #1 and #2 respectively. (**B**) Phosphopeptide map of endogenous 14-3-3σ precipitated from MCF10A cells. Treatment with TGFβ1 and directions of electrophoresis and chromatography are indicated. (**C**) Elution positions of ^32^P-labled amino acids upon Edman degradation are shown for phosphopeptides #1 and #2, respectively. Corresponding tryptic peptide is aligned below the panels. (**D**) Phosphopeptide maps of the wild-type and Ser74Ala (S74A), Ser69Ala (S69A) and Ser69, 74Ala (S69, 74A) mutants of 14-3-3σ are shown. Disappearance of the spots corresponding to the migration positions of phosphopeptides #1 and #2 are indicated by the arrows. (**E**) Ser69 and Ser74 residues are located within N-terminal part of 14-3-3σ in close proximity to CDK binding sequence AA**WRVL**SS (residues 57–64) [51] and to the basic cluster in the conserved amphipathic groove, consisting of K49, R56 and R127, and mediating the interaction of 14-3-3 with the phosphoamino acid in its ligands [38]. The image shows the structure of the p53 C-terminus bound to 14-3-3σ [52], PDB entry 3LW1.

As our data indicate that 14-3-3σ may be phosphorylated on multiple sites, we performed two-dimensional phosphopeptide mapping which allows monitoring of all phosphopeptides and the level of ^32^P incorporation in these peptides (Figure 3). We found that TGFβ1 induced phosphorylation of two phosphopeptides of ectopically expressed 14-3-3σ, indicated as phosphopeptides #1 and #2 (Figure 3A). The same phosphopeptides were observed in endogenous 14-3-3σ in TGFβ1-treated MCF10A cells (Figure 3B). To identify the sites of phosphorylation, TGFβ1-regulated phosphopeptides were subjected to radiochemical sequencing and to phosphoamino acid analysis. We found that the phosphopeptide #1 was strongly phosphorylated at position 6, and the phosphopeptide #2 showed two sites of phosphorylation at positions 1 and 6 (Figure 3C). Alignment of possible tryptic peptides showed that the peptide Ser69-Lys77 has serine residues at positions 1 and 6. Ser69 and Ser74 were mutated to alanine residues to abrogate phosphorylation at these sites. 14-3-3σ with mutated Ser69 and Ser74 did not show TGFβ1-dependent induction of phosphorylation, as compared to the wild-type construct (Figure S6). Two-dimensional phosphopeptides maps of mutated 14-3-3σ showed disappearance of phosphopeptides #1 for Ser74Ala mutant, phosphopeptides #2 for Ser69Ala mutant, and both phosphopeptides for the double Ser69/74Ala mutant (Figure 3D). The peptide sequence around Ser69 and Ser74 residues showed similarity to the Casein Kinase-2 (CK2) consensus [17]. Co-expression of CK2α with the wild-type 14-3-3σ led to enhancement of 14-3-3σ phosphorylation, as quantified by ^32^P incorporation in peptides #1 and #2 (Figure S7). Thus, we have identified Ser69 and Ser74 as the sites of TGFβ1-dependent phosphorylation. Ser69 and Ser74 residues are located within amino-terminal domain of 14-3-3σ protein (Figure 3E). Crystal structure and mutational studies showed that 14-3-3σ can bind to other proteins through residues located at the both, amino- and carboxy-terminal parts [18], [19]. These findings point to possibility of involvement of Ser69 and Ser74 residues in the interaction of 14-3-3σ and its binding partners.

### Phosphorylation of 14-3-3σ is a feed-forward mechanism in Smad3-dependent transcription

**Figure 4:**
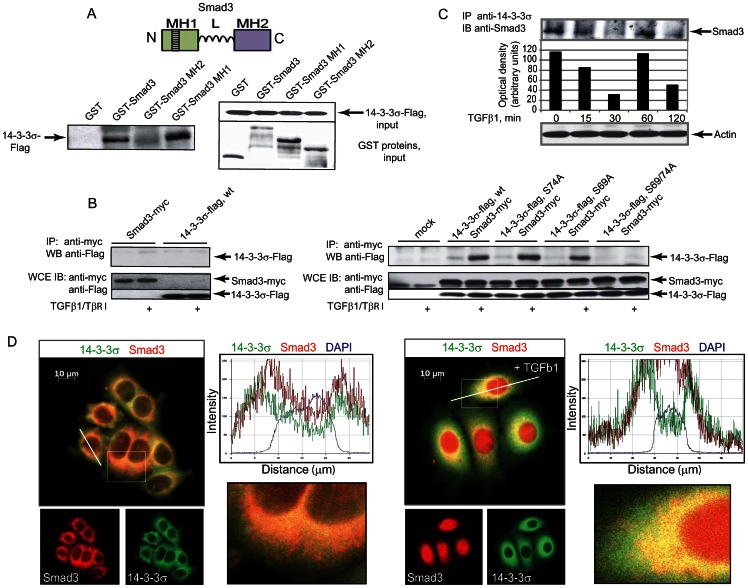
14-3-3σ forms a complex with Smad3. (**A**) 14-3-3σ interacts with Smad3 *in vitro*. The interaction is mediated by the MH1 domain. A diagram of Smad3 with its three domains MH1, linker (L) and MH2 is shown on the top panel. N and C denote the amino and carboxyl termini. The left panel shows co-precipitated 14-3-3σ, the right panels show inputs of 14-3-3σ (upper part) and GST constructs (lower part). (**B**) Complex formation between Smad3 and 14-3-3σ depends on phosphorylation at Ser69 and Ser74. HEK293T cells were transfected with protein expressing DNA constructs as indicated. Migration position of co-precipitated 14-3-3σ is shown by the arrow. The levels of expression of 14-3-3σ and Smad3 proteins are shown on the lower panels. (**C**) Treatment of cells with TGFβ1 modulates interaction between endogenous Smad3 and 14-3-3σ in time dependent manner. MCF7 cells were treated with TGFβ at concentration of 5 ng/ml for the indicated times. Cell lysates were immunoprecipitated with anti-14-3-3σ antibody. Anti-Smad3 antibody was used in Western blot analysis. Densitometry analysis of Smad3 immunoblot is shown in the accompanying graph. (**D**) Co-localization of 14-3-3σ and Smad3 after treatment of the cells with TGFβ at concentration of 5 ng/ml. Images of immunofluorescent staining of the cells overexpressing 14-3-3σ and Smad3 proteins, are shown. The magnified cells and the line traces are shown on the right.

14-3-3σ is known to act as a scaffold by interacting with over 200 target proteins in phosphoserine-dependent and phosphoserine-independent manners [15], [20]. We observed that the wild-type 14-3-3σ interacted with the full-length and the MH1 domain of Smad3 in GST pull-down assay (Figure 4A). We observed that the interaction between Smad3 and wild-type 14-3-3σ was induced by treatment of the cells with TGFβ1 and co-transfection with constitutively active TβR-I (Figure 4B). Abrogation of 14-3-3σ phosphorylation at Ser69 and Ser74 completely blocked the interaction, while single Ser69Ala and Ser74Ala mutants were able to form a complex with Smad3. We showed that treatment of cells with TGFβ1 modulates interaction between endogenous Smad3 and 14-3-3σ in time dependent manner. Moreover, this interaction correlates with profile of 14-3-3σ phosphorylation at Ser69 and Ser74 residues (Figure 4C). We also observed co-localization of Smad3 and wild-type 14-3-3σ in cells using immunofluorescence staining (Figure 4D). Thus, the interaction of Smad3 and 14-3-3σ requires phosphorylation on Ser69 and Ser74 in 14-3-3σ.

**Figure 5:**
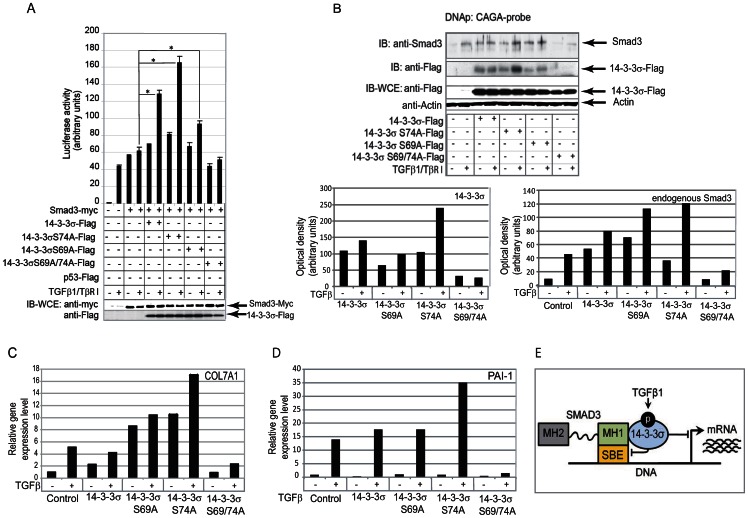
Smad3-dependent transcriptional activation is regulated by phosphorylation of 14-3-3σ at Ser69 and Ser74. (**A**) CAGA (12)-luc reporter activation upon expression of 14-3-3σ constructs and Smad3, * - p value<0.05 (**B**) DNA precipitation assay shows the recruitment of Smad3 to the CAGA element in the present of wild-type and mutant 14-3-3σ proteins. Expression controls for Smad3 and 14-3-3σ constructs are shown in two lower panels. Densitometry analysis of the protein precipitation is shown in the accompanying graphs. (**C**, **D**) TGFβ dependent and Smad3 governed transcriptional activation of PAI-1 and COL7A1 genes. For gene expression analysis, MCF10A cells were stably transfected with the constructs expressing wild type or mutated 14-3-3σ protein and treated with TGFβ1 at concentration of 5 ng/ml for 12 hours. The data shown correspond to a representative experiment out of three performed. (**E**) Schematic presentation of the restrictive role of 14-3-3σ phosphorylation on TGFβ1/Smad3-dependent transcription.

Smad3 is a transcription factor which binds directly to a specific promoter element CAGA [21]. Thereby, we explored whether phosphorylation and interaction of 14-3-3σ with Smad3 regulates TGFβ/Smad3-dependent transcriptional activation of CAGA (12)-luc luciferase reporter (Figure 5A). Our data suggest that overexpression of the wild type or a single Ser74 mutant 14-3-3σ protein could significantly increase TGFβ and Smad3-dependent transactivation activity of the reporter (up to 2 folds). However, the abrogation of TGFβ dependent phosphorylation at Ser69 and Ser74 abolished the ability of 14-3-3σ to co-activate TGFβ and Smad3-dependent transcription.

Analysis of proteins bound to CAGA element (DNA precipitation assay) showed that the presence of Smad3 and its upper migrating (phosphorylated) form correlated with stimulatory effect on transcriptional activity (Figure 5B). No detection of the double mutant of 14-3-3σ also correlated with decreased transcriptional activation. Moreover, TGFβ dependent transactivation of Smad3 responsive genes PAI-1 and COL7A1, possibly through CAGA box elements in their promoters, correlated with involvement of 14-3-3σ in the transcriptional complex with endogenous Smad3, and confirms importance of phosphorylation of 14-3-3σ at Ser69 and Ser74 for tuning of Smad3-dependent transcriptional activation (Figure 5C, D).

Thus, TGFβ-dependent phosphorylation of 14-3-3σ is a feed-forward mechanism for TGFβ/Smad3 transcriptional regulation. The feed-forward tuning included 14-3-3σ recruitment to the Smad3-initiated transcriptional complex, which depends on the phosphorylation state of 14-3-3σ at Ser69 and Ser74 (Figure 5E).

### TGFβ1-dependent phosphorylation of 14-3-3σ regulates MCF7 tumor progenitor population

A large body of evidence has demonstrated that many human tumors contain a heterogeneous mixture of different cell types and are maintained by a small cell population called cancer stem cells (CSC) or tumor progenitors, which is responsible for tumor formation and metastasis, and implicated to therapy resistance [22]. TGFβ signaling has been implicated in the maintenance of both normal somatic stem cells and cancer stem cells. TGFβ axis plays a dual role in cancer progression switching from tumor suppression in early stages of cancer to promoting invasion and metastasis at later stages. Therefore, TGFβ may have either a CSC-suppressing or CSC-promoting functions depending on cellular context.

**Figure 6:**
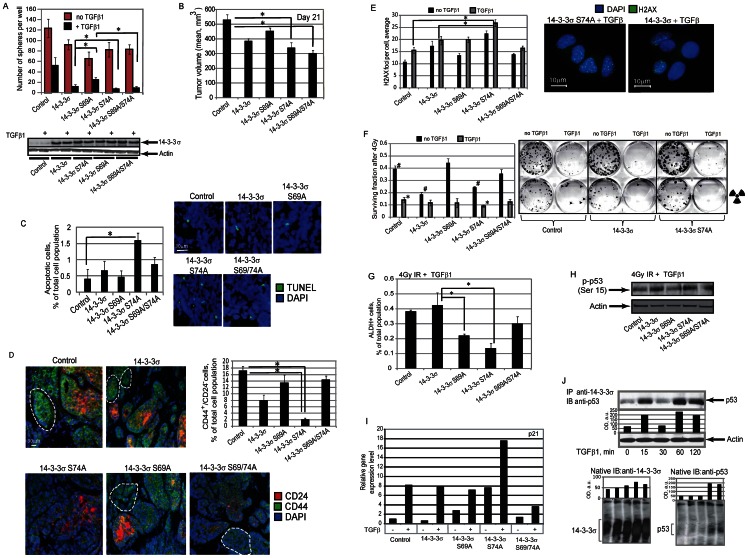
TGFβ1-dependent 14-3-3σ phosphorylation plays a role in regulation of tumor progenitor population. (**A**) The role of TGFβ1-dependent 14-3-3σ phosphorylation on TGFβ1-mediated inhibition of sphere initiating population in MCF7 cells. MCF7 cells were stably transfected with the constructs expressing wild type or mutated 14-3-3σ proteins, along with control vector, and used for sphere formation assays in the presence of TGFβ1 at concentration of 5 ng/ml; * - p-value<0.05. (**B**) Tumorigenic properties of MCF7 cells stably expressing wild type or mutated 14-3-3σ proteins, along with control vector, were analyzed *in vivo* using NOD/SCID mouse xenograft model. Each experimental group contained at least five mice. Graph shows tumor volumes at day 21 after cell injection; * - p value<0.05. (**C**) Analysis of cell apoptosis in the xenograft tumors using TUNEL staining. Cells in at least 3 randomly selected fields of view were counted for each condition; * - p-value<0.05. (**D**) Representative fluorescent images of CD44 and CD24 co-immunostaining in MCF7 xenograft tumors. The percentage of CD44+/CD24− tumor progenitor cells (outlined in white) was counted in at least 3 randomly selected fields of view for each condition; * - p-value<0.05. Scale bar, 30 µm. (**E**) Immunofluorescence detection of phosphorylated γ-H2A.X at 4 hours after irradiation. MCF7 cells were stably transfected with the constructs expressing wild type or mutated 14-3-3σ proteins, along with control vector, pre-treated with TGFβ1 at concentration of 5 ng/ml for 12 hours and irradiated with 4 Gy X-ray dose. For quantification, at least 100 cells per condition were counted; * - p-value<0.05. Scale bar, 10 µm. (**F**) Clonogenic radiation survival assay. MCF7 cells were stably transfected with the constructs expressing wild type or mutated 14-3-3σ proteins, along with control vector, pre-treated with TGFβ1 at concentration of 5 ng/ml for 12 hours and irradiated with 4 Gy X-ray dose; * - p-value<0.05. (**G**) Flow cytometry analysis of tumor progenitor population using ALDEFLUOR assay. MCF7 cells were stably transfected with constructs expressing wild type or mutated 14-3-3σ proteins, along with control vector, treated with TGFβ1 at concentration of 5 ng/ml for 7 days, irradiated with 4 Gy X-ray dose, and analyzed 3 days later; * - p-value<0.05. Representative experiments out of 3 performed are shown. (**H**) Western blot analysis of p53 phosphorylation at Ser15 in MCF7 cells stably transfected with constructs expressing wild type or mutated 14-3-3σ proteins, along with control vector, treated with TGFβ1 at concentration of 5 ng/ml for 12 h and irradiated with 4 Gy X-ray dose. The cells were analyzed 4 hours after irradiation. (**I**) Results of semi-quantitative RT-PCR analysis for p21 gene expression in MCF7 cells stably transfected with DNA constructs encoding wild type or mutated 14-3-3σ proteins, along with control DNA plasmid and treated with TGFβ1 at concentration of 5 ng/ml for 12 h. (**J**) Treatment of the cells with TGFβ1 modulates interaction between endogenous p53 and 14-3-3σ in time dependent manner. MCF7 cells were treated with TGFβ1 at concentration of 5 ng/ml for the indicated times. Cell lysates were immunoprecipitated with anti-14-3-3σ antibody. Anti-p53 antibody was used for immunoblot analysis. The results of blue native PAGE are shown below.

In order to explore the impact of TGFβ dependent 14-3-3σ phosphorylation on the maintenance of cancer progenitor population, we analyzed the MCF7 cells overexpressing 14-3-3σ wild type or mutant proteins in sphere formation assay. We found that activation of TGFβ signaling reduced the sphere formation properties of MCF7 cells in the manner dependent on 14-3-3σ phosphorylation. Notably that overexpression of the wild type 14-3-3σ and mutant protein 14-3-3σ Ser74Ala and 14-3-3σ Ser69/74Ala had significantly higher impact on TGFβ dependent inhibition of sphere initiating cells than expression of 14-3-3σ Ser69Ala mutant protein suggesting that TGFβ1 dependent phosphorylation of 14-3-3σ at Ser 69 and Ser 74 can play a different role in regulation of CSCs by TGFβ1 activation (Figure 6A). Despite the marginal effect of the double mutant 14-3-3σ Ser69/74Ala protein on the Smad3-dependent transcription, its overexpression significantly inhibits the putative CSC population even without disturbing the Smad3-dependent transcription. This observation can potentially suggest that abrogation of TGFβ1 dependent phosphorylation of 14-3-3σ at two sites can trigger the Smad3 independent mechanisms of CSC regulation by TGFβ1 axis.

Tumorigenic properties of MCF7 cells stably expressing wild type and the mutant 14-3-3σ proteins were analyzed *in vivo* using NOD/SCID mouse xenograft models. Our data indicates that the MCF7 xenograft tumors expressing 14-3-3σ Ser74Ala and 14-3-3σ Ser69/74Ala mutant proteins showed statistically significant decrease in the growth rate *in vivo* compared with a control group (Figure 6B). Analysis of cell apoptosis in the xenograft tumors using TUNEL staining confirmed a higher number of apoptotic cells in the MCF7 xenograft tumors expressing 14-3-3σ Ser74Ala mutant protein (Figure 6C). This result is in agreement with *in vitro* data for TGFβ dependent inhibition of sphere formation (Figure 6A) and suggests that phosphorylation of 14-3-3σ at Ser69 plays an important role in cell tumorigenicity and survival.

In breast cancers, a subset of the cells with high aldehyde dehydrogenase (ALDH1) activity and CD44^+^/CD24^−/low^ phenotype has been characterized as CSCs population with tumorigenic and radioresistant phenotype [23], [24]. Histological analysis of MCF7 xenograft tumors expressing 14-3-3σ wild type or Ser74Ala mutant proteins revealed a decrease in CD44^+^/CD24^−/low^ cell phenotype as compared with a control group or with the MCF7 xenograft tumors expressing 14-3-3σ Ser69Ala or double mutant proteins (Figure 6D).

As suggested by preclinical studies and clinical observation, resistance to conventional therapy, including irradiation, has been reported to be a defining characteristic of CSCs from various tumor types, including breast cancer [25]. One of the best-characterized chromatin modification events in responses to cell irradiation is histone H2A.X phosphorylation by ATM or ATR serine/threonine protein kinase. The phosphorylated form of H2A.X (γ-H2A.X) forms nuclear foci on the sites of DNA double strand breaks (DSB) and the number of γ-H2AX foci approximates the number of DSBs induced [26]. Recent studies demonstrated that in murine mammary gland CSCs, γ-H2A.X foci resolved faster than in non-CSC population that perhaps is reflective of more efficient DSB repair in CSCs [27]. Immunofluorescent detection of phosphorylated γ-H2A.X revealed more residual DNA damage at 4 hours after irradiation in the cell overexpressing 14-3-3σ Ser74Ala mutant protein and treated with TGFβ as compared to the control cells (Figure 6E). Because successful DNA reparation in the irradiated cells is associated with their survival, we further assessed the surviving fraction of the cells treated or non-treated with TGFβ and irradiated with 4 Gy X-ray dose. Our data suggest that activation of TGFβ signaling pathway decreases cell survival after irradiation and, besides of that, surviving fraction of the cells overexpressing 14-3-3σ Ser74Ala mutant protein was significantly reduced as compared to the control cells (Figure 6F). These results showed an impaired repair capability in the cells overexpressing 14-3-3σ Ser74Ala mutant after TGFβ treatment, which is consistent with the decreased cell survival.

Preclinical assays and clinical findings suggest that a higher proportion of tumor initiating cells correlates with higher tumor radioresistance [28]. ALDH activity selectively defines an enhanced tumor-initiating cell population in human breast tumors and cancer cell lines, including MCF7 cells [23], [29]. Flow cytometry analysis of the cells irradiated with 4 Gy and treated with TGFβ1 revealed significantly lower ALDH+ tumor progenitor population in the cells overexpressing 14-3-3σ Ser74Ala mutant protein when compared to other cell types (Figure 6G).

TP53 (p53) has been recognized as an important tumor suppressor protein, functioning mainly through transcriptional control of target genes that influence cell response to irradiation. Ser15 phosphorylation is a critical event in transcriptional activation of p53 and results in upregulation of downstream target protein p21, which accumulation results in inhibition of CSC population and tumor radiosensitization [30], [31]. Our data suggest that cells overexpressing 14-3-3σ Ser74Ala mutant protein have high level of p53 phosphorylation at Ser 15 and transcription upregulation of p21 in response to TGFβ1 treatment, as compared to the cells overexpressing wild type of 14-3-3σ or mutant 14-3-3σ proteins (Figure 6 H, I).

Previous studies have shown that 14-3-3σ interacts with p53 directly [32]. We found that treatment of the cells with TGFβ1 modulated interaction between endogenous p53 and 14-3-3σ in time-dependent manner (Figure 6J). This interaction correlated with the profile of 14-3-3σ phosphorylation at Ser69 and Ser74 residues (Figure 2E). To identify physiological 14-3-3σ–p53 interactions, we performed the blue native PAGE. Consistent with the immunoprecipitation results, we observed 14-3-3σ–p53 protein complex formation in time-dependent manner, which correspond to the profile of the TGFβ1-dependent 14-3-3σ phosphorylation (Figure 6J). These results indicate that transcriptional regulation of p53 and interaction with 14-3-3σ can be governed by TGFβ dependent 14-3-3σ phosphorylation.

### 14-3-3σ–p53 protein complexes play a role in the regulation of Smad3 dependent transcription

**Figure 7:**
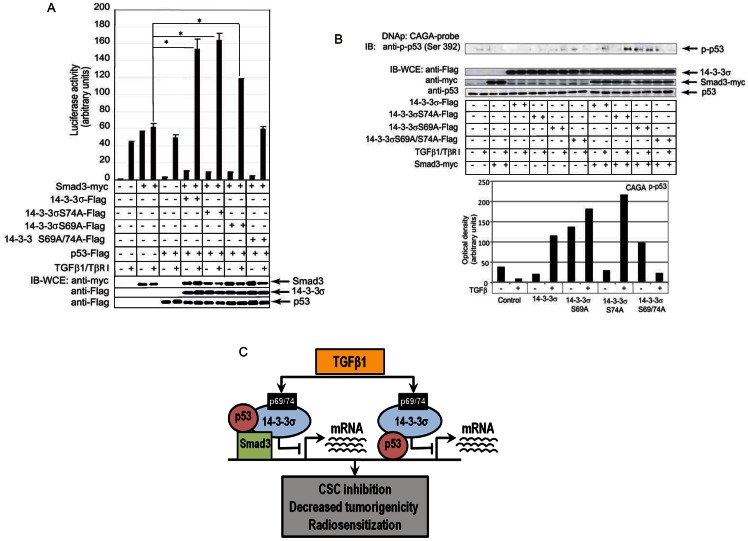
TGFβ1-dependent phosphorylation of 14-3-3σ regulates Smad3 dependent transcription through involvement of p53 in the transcriptional complexes. (**A**) Results of the luciferase activity assay using CAGA (12)-luc reporter. Co-expression of p53 had a restrictive effect on Smad3-dependent transcriptional activity in cells not treated with TGFβ, * - p value<0.05. (**B**) The results of DNA immunoprecipitation analysis. p53 phosphorylated at Ser392 is recruited to the CAGA element in presence of 14-3-3σ and Smad3 in the manner dependent on 14-3-3σ phosphorylation. Densitometry analysis of the p53 precipitation is shown in accompanying graph. (**C**) Schematic presentation of the role of 14-3-3σ phosphorylation on a functional interaction of TGFβ1/Smad3 and p53/p21 signaling.

Previous reports indicated that wild-type p53 may associate with SMAD2 and SMAD3 in a TGF-β-dependent manner, although the exact molecular mechanism of such association has not been reported [33]. We analyzed if 14-3-3σ–p53 protein complexes can be involved in the regulation of Smad3 transcriptional activity. Our study demonstrated that co-expression of p53 had a restrictive effect on Smad3-dependent transcriptional activity in cells not treated with TGFβ (Figure 7A). However, upon treatment with TGFβ1 the level of Smad3-dependent transcription was similar for the control cells and the cells expressing p53 protein of either ectopic or endogenous origin (Figure 5A, Figure 7A).

To further confirm the involvement of p53 in Smad3- regulated transcription, we analyzed the recruitment of p53 phosphorylated at Ser392 to the CAGA box element. Phosphorylation on COOH-terminal Ser392 enhances the specific DNA binding of p53 *in vitro* [34]. DNA precipitation experiments showed that Ser392-phosphorylated p53 can be recruited to the CAGA element in presence of the wild-type or mutants of 14-3-3σ and Smad3 in TGFβ dependent manner. The level of p53 protein bound to the CAGA box in the presence of ectopically expressed Smad3 and 14-3-3σ proteins was remarkably higher when phosphorylation of 14-3-3σ at Ser69 or Ser74 was abrogated. Double mutant Ser69/74Ala of 14-3-3σ decreased recruitment of p53 to the CAGA box element (Figure 7B). Notably that involvement of phospho-p53 in the Smad3/14-3-3σ/CAGA complex positively correlated with expression of Smad3-responsive genes PAI and COL7A1 suggesting that 14-3-3 phosphorylation could be one of the mechanism regulating cooperation between p53 and TGFβ signaling (Figure 7B, Figure 5 C, D).

Thus, TGFβ-dependent phosphorylation of 14-3-3σ is a feed-forward mechanism for TGFβ/Smad3 transcriptional regulation. The feed-forward tuning includes a 14-3-3σ-dependent recruitment of p53 to a Smad3-initiated transcriptional complex, which led to restriction of ligand-independent transcription and to enhancement of the ligand-induced effect. This indicates that p53 is an enhancer of bi-stability for Smad3-dependent transcriptional activation, e.g. on-off accentuation. Taken together, these results suggest that TGFβ dependent 14-3-3σ phosphorylation plays an important role in regulation of tumorigenicity and radioresistance that can be mediated by the integrated TGFβ and p53/21 signaling pathways (Figure 7C).

## Discussion

Signaling by a network, as compared to the model of unidirectional signaling pathways [35], is required for coordination of various functions in cells undergoing significant changes, e.g. proliferation or carcinogenic transformation. Our study identified proteins which may mediate coordinated regulation of cell signaling and metabolism, proliferation, death and differentiation of human breast epithelial cells. The network built with TGFβ-regulated phosphoproteins showed characteristics of a scale-free network. However, the frequency of highly-connected hubs is higher than would be expected according to a power law distribution of connections in an ideal scale-free network. The presence of such hubs indicates the key points of convergence for various signals. In the TGFβ phosphoprotein-network, these hubs represent signaling activities initiated and/or mediated by EGF, TNF, IGF, AKT, Src, H-Ras, CDK2 and NF-kB. Therefore the status of these hubs may dictate the output of TGFβ action on cells. For many of the above mentioned hubs, functional cross-talks with TGFβ have been reported in model systems specific for each of the hubs [1], [2], [3]. Phosphoproteome profiling of TGFβ1 in MCF7 cells using *in vivo* metabolic labeling with ^32^P was the first step in an exploration of TGFβ phosphoproteome. Despite use of different cell lines and different technical approaches, we identified in MCF10A cells some of the proteins which were regulated by TGFβ1 in MCF7 cells, e.g. keratin 10, enolase-1 and HSP70 [36]. IMAC-enrichment of phosphoproteins showed capability to enrich for low abundance proteins, which explains high representation of regulatory proteins. Thus, our approach provided the most comprehensive description of phosphorylation events initiated by TGFβ1.

To confirm that our network-based approach unveils novel crucial regulatory mechanisms, we explored the role of TGFβ1-dependent phosphorylation of 14-3-3σ. Some phosphorylation sites in 14-3-3σ important for its function were identified earlier, e.g. Thr198, Ser216, Thr291, Ser428, Ser642 [37], [38]. In this report, we identified two novel and TGFβ-dependent phosphorylation sites, i.e. Ser69 and Ser74. Identified by us network suggested that phosphorylation of 14-3-3σ at Ser69 and Ser74 may play crucial role in TGFβ signaling. Indeed, we observed that 14-3-3σ phosphorylation is a feed-forward mechanism in TGFβ/Smad3-dependent transcription. Our data suggest that overexpression of the wild type or single Ser74 mutant 14-3-3σ proteins could significantly increase TGFβ and Smad3-dependent transcription activity of the reporter. In addition to the regulation of Smad3-specific transcription, 14-3-3σ phosphorylation at Ser69 and Ser74 regulates the CSC-like features of breast cancer cells.

Recent studies indicate that a subset of cancer cells referred to as cancer stem cells play a critical role in tumor initiation and resistance to anticancer therapy. The tumor cell populations surviving chemo- and radiotherapy are enriched for CSCs and have the phenotypic hallmarks of epithelial-to-mesenchymal transition (EMT) [39]. The acquisition of EMT program is a critical process for the progression of cancers from local carcinomas to invasive malignancies, which is often associated with the loss of epithelial differentiation and gain of mesenchymal phenotype. A growing body of evidence has demonstrated a molecular link between EMT and self-renewal, suggesting that EMT programs play critical roles in the maintenance and generation of CSCs. Recent studies showed that breast cancer cells undergoing EMT gain CSC properties including the ability to self-renew, tumorigenicity and expression of the CSC phenotype CD44^+^/CD24^−/low^ [40].

TGFβ signaling was found to be a potent inducer of EMT that may enhance cancer progression by dedifferentiation of non-CSCs into tumorigenic and invasive CSCs [41], [42]. However, in contrast to reports demonstrating that TGFβ induces expansion of CSCs and promotes tumor growth, TGFβ signaling has also been shown to suppress tumorigenesis and reduce the number of CSCs through differentiation in the breast epithelial cell lines derived from MCF10A cells and in clinical breast cancer samples [43]. Recent observations suggest that TGFβ inhibits the sphere-initiating CSC population in the MCF7 breast cancer cells [44]. These controversial results may suggest a complex role of TGFβ in the regulation of CSCs from the different tumors that may be due to the differential expression of TGFβ regulators and effectors in the distinct molecular subtypes of breast cancers. In support of this hypothesis, Kumar and coauthors demonstrated that tissues transglutaminase (TG2) is a downstream effector of TGFβ-induced EMT, and TGFβ signaling itself failed to induce EMT, CSC phenotype and drug resistance in the cells lacking TG2 expression, including MCF10A and MCF7 cells [45]. Another study confirmed that TGFβ signaling induces the formation of tumor-initiating cells only in claudin^low^ breast cancer cell lines, but not in MCF7 cells [46]. We found that activation of TGFβ signaling reduced the sphere formation properties of MCF7 cells in the manner dependent on 14-3-3σ phosphorylation. Notably that TGFβ-mediated inhibitory effect of 14-3-3σ on putative CSC population inversely correlated with an effect of TGFβ-dependent 14-3-3σ phosphorylation on Smad3-dependent transcription. The overexpression of the wild type 14-3-3σ and mutant 14-3-3σ Ser74Ala proteins had significantly higher impact on TGFβ dependent inhibition of putative CSC population than expression of 14-3-3σ Ser69Ala mutant protein suggesting that TGFβ1 dependent phosphorylation of 14-3-3σ at Ser 69 and Ser 74 can play a different role in regulation of CSCs by TGFβ1 activation. It is noteworthy that overexpression of the double mutant 14-3-3σ Ser69/74Ala protein has an inhibitory effect on the putative CSC population even without disturbing the Smad3-dependent transcription. This observation can potentially suggest that abrogation of TGFβ1 dependent phosphorylation of 14-3-3σ at two sites can results in the involvement of 14-3-3σ in the alternative, Smad3 independent routes of CSC regulation via TGFβ1 signaling network.

Resistance to radiation therapy has been reported to be a defining characteristic of CSCs from various tumor types, including breast cancer. One of the potential mechanisms of a high radioresistance of CSC population is activation of the DNA damage response. The phenotypic radiation resistance of CD44^+^/CD24^−^ breast CSC population has been attributed to the enhanced activation of ATM signaling that is one of the key molecular mechanisms by which somatic cells respond to DNA damage [47]. ATM and ATR protein kinases are activated upon ionized radiation-induced DNA double strand breaks and phosphorylate p53 tumor suppressor protein on Ser 15 [48], [49]. Ser15 phosphorylation is a critical event in transcriptional activation of p53 and results in upregulation of downstream target protein p21 [30]. p21 is an universal inhibitor of cyclin dependent kinases, which mediates p53 induced growth arrest at G1 and G2 phases of cell cycle [50]. Recent studies have suggested an important role of p21 in regulating the features of cancer stem cells [25]. Ectopic expression of p21 repressed EMT, stem cell marker expression, self-renewal capacity and tumorigenicity of breast cancer cells. Moreover, accumulation of p21 protein mediated by blockage of its degradation results in radiosensitization of human breast cancer cells, including MCF7 cells [31]. Our data suggest that cells overexpressing 14-3-3σ Ser74Ala mutant protein increases p53 phosphorylation at Ser 15 and transcription upregulation of p21 in response to TGFβ1 treatment, as compared to the cells overexpressing wild type of 14-3-3σ or other mutant 14-3-3σ proteins. These results are consistent with our data demonstrating an impaired repair capability and decreased cell survival in the cells overexpressing 14-3-3σ Ser74Ala mutant after TGFβ treatment.

Previous studies have shown that 14-3-3σ interacts with p53 directly [32]. We further confirmed that TGFβ1-mediated phosphorylation modulated interaction between 14-3-3σ protein and p53, which in turn regulates the involvement of p53 in Smad3-dependent transcription. The 14-3-3σ-dependent recruitment of p53 to a Smad3-initiated transcriptional complex led to restriction of ligand-independent transcription and to enhancement of the ligand-induced effect.

Taken together these results suggest that TGFβ1-dependent phosphorylation of 14-3-3σ is a scaffold for tuning of TGFβ cellular response that orchestrates a functional interaction of TGFβ/Smad3 and p53/21 signaling pathways, regulates tumorigenic and radioresistant cancer cell population and can provide a new potential target for anticancer therapy.

## Supporting Information

Figure S1 **Representative 2D gel of Fe-IMAC enriched phosphoproteins of MCF10A cells treated with TGFβ1 for 2 h.** Direction of isoelectrofocusing is indicated on the top of the gel image. Migration positions of proteins regulated by TGFβ1 are indicated by lines, with annotation of proteins as in Table 1.
(TIF)

Figure S2 **Representative 2D gels of Fe-IMAC enriched phosphoproteins of MCF-10A cells treated with TGFβ1 for indicated time periods are shown.** Directions of isoelectrofocusing are indicated on the top of the gel image. Migration positions of molecular mass markers upon SDS-PAGE are indicated on the side of the image.
(TIF)

Figure S3 **Validation of phosphorylation of identified proteins.** Protein spots corresponding to (**A**) FKBP12, (**B**) Actin and (**C**) Enolase1 are shown with quantification of relative optical density. (**D**) Phosphorylation of these proteins was monitored by immunoprecipitation with anti-pSer/pThr/pTyr and immunoblotting with specific antibodies, as indicated. Loading control is shown in accompanying panel.
(TIF)

Figure S4 **Functional and dynamic clustering of TGFβ1-regulated phosphoproteins.** (**A**) Functional clusters and number of proteins assigned to the clusters are indicated. (**B**) Heatmap of the TGFβ1-regulated phosphoproteins clustered according to the changes in their expression. (**C**) Dynamics of phosphoproteins involved in regulation of cell proliferation and cell death. Proteins are annotated in Gene Ontology (GO) terms.
(TIF)

Figure S5 **Network of TGFβ1-regulated phosphoproteins.** TGFβ1-regulated proteins are presented in a network with their known targets and regulators. Strings between proteins/species represent dependencies which describe physical and/or functional interactions between these species.
(TIF)

Figure S6 (TIF)

Figure S7 **Two-dimensional phosphopeptides mapping showed increase of the phosphorylation of two 14-3-3σ peptides in response to CK2 overexpression.** 293T cells were transfected with CK2 expressing construct or control vector and subjected to the two-dimensional phosphopeptides mapping analysis. Migration positions of these phosphopeptides are shown by arrows, as #1 and #2 respectively.
(TIF)
