# Supplementary figures and images for: A Role of TGFß1 Dependent 14-3-3σ Phosphorylation at Ser69 and Ser74 in the Regulation of Gene Transcription, Stemness and Radioresistance
Source: PLoS One. 2013 May 31;8(5):e65163. doi: 10.1371/journal.pone.0065163 (PMC3669286; doi:10.1371/journal.pone.0065163)

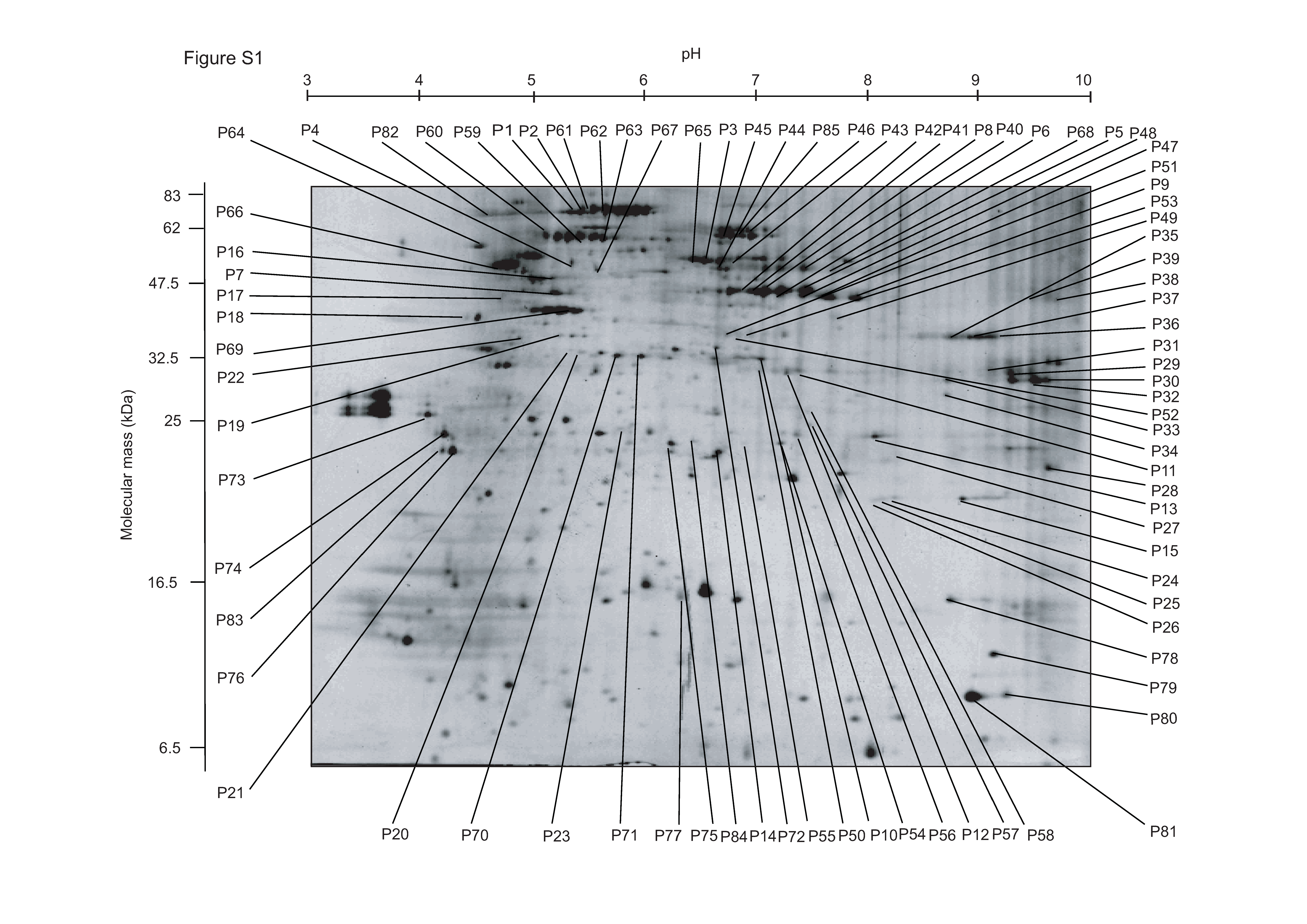

Supplement: Figure S1 — Representative 2D gel of Fe-IMAC enriched phosphoproteins of MCF10A cells treated with TGFβ1 for 2 h. Direction of isoelectrofocusing is indicated on the top of the gel image. Migration positions of proteins regulated by TGFβ1 are indicated by lines, with annotation of proteins as in Table 1. (TIF) [file pone.0065163.s001.tif]

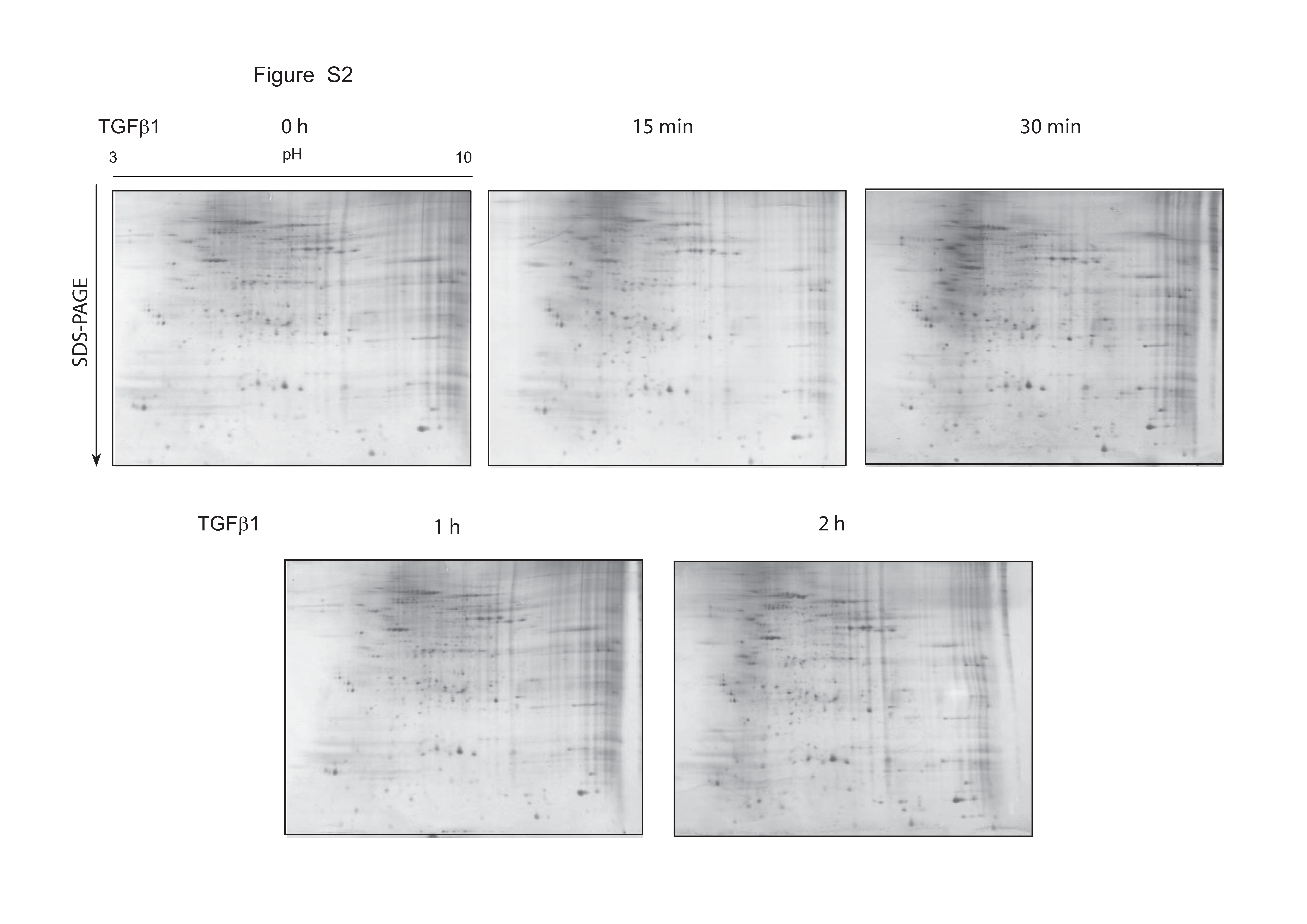

Supplement: Figure S2 — Representative 2D gels of Fe-IMAC enriched phosphoproteins of MCF-10A cells treated with TGFβ1 for indicated time periods are shown. Directions of isoelectrofocusing are indicated on the top of the gel image. Migration positions of molecular mass markers upon SDS-PAGE are indicated on the side of the image. (TIF) [file pone.0065163.s002.tif]

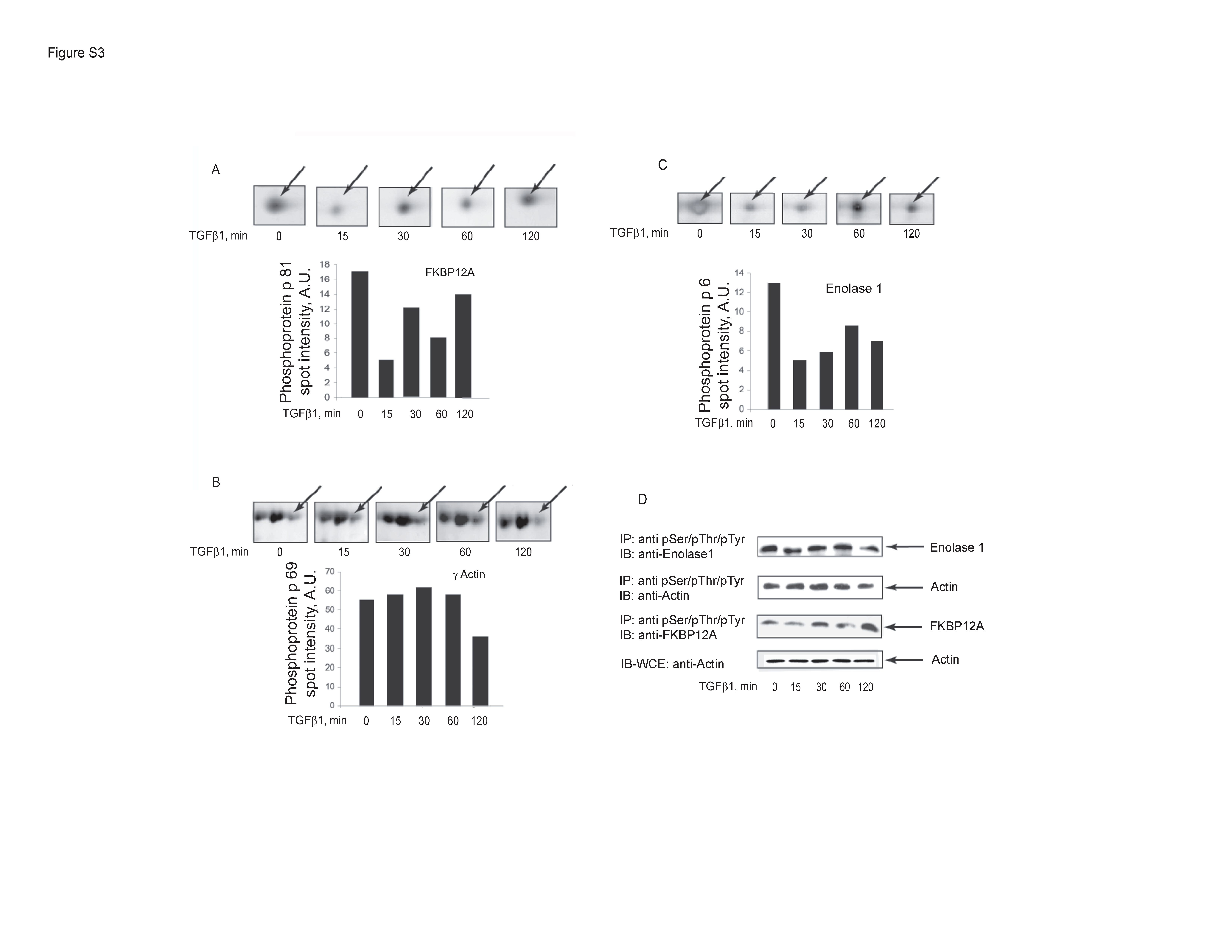

Supplement: Figure S3 — Validation of phosphorylation of identified proteins. Protein spots corresponding to (A) FKBP12, (B) Actin and (C) Enolase1 are shown with quantification of relative optical density. (D) Phosphorylation of these proteins was monitored by immunoprecipitation with anti-pSer/pThr/pTyr and immunoblotting with specific antibodies, as indicated. Loading control is shown in accompanying panel. (TIF) [file pone.0065163.s003.tif]

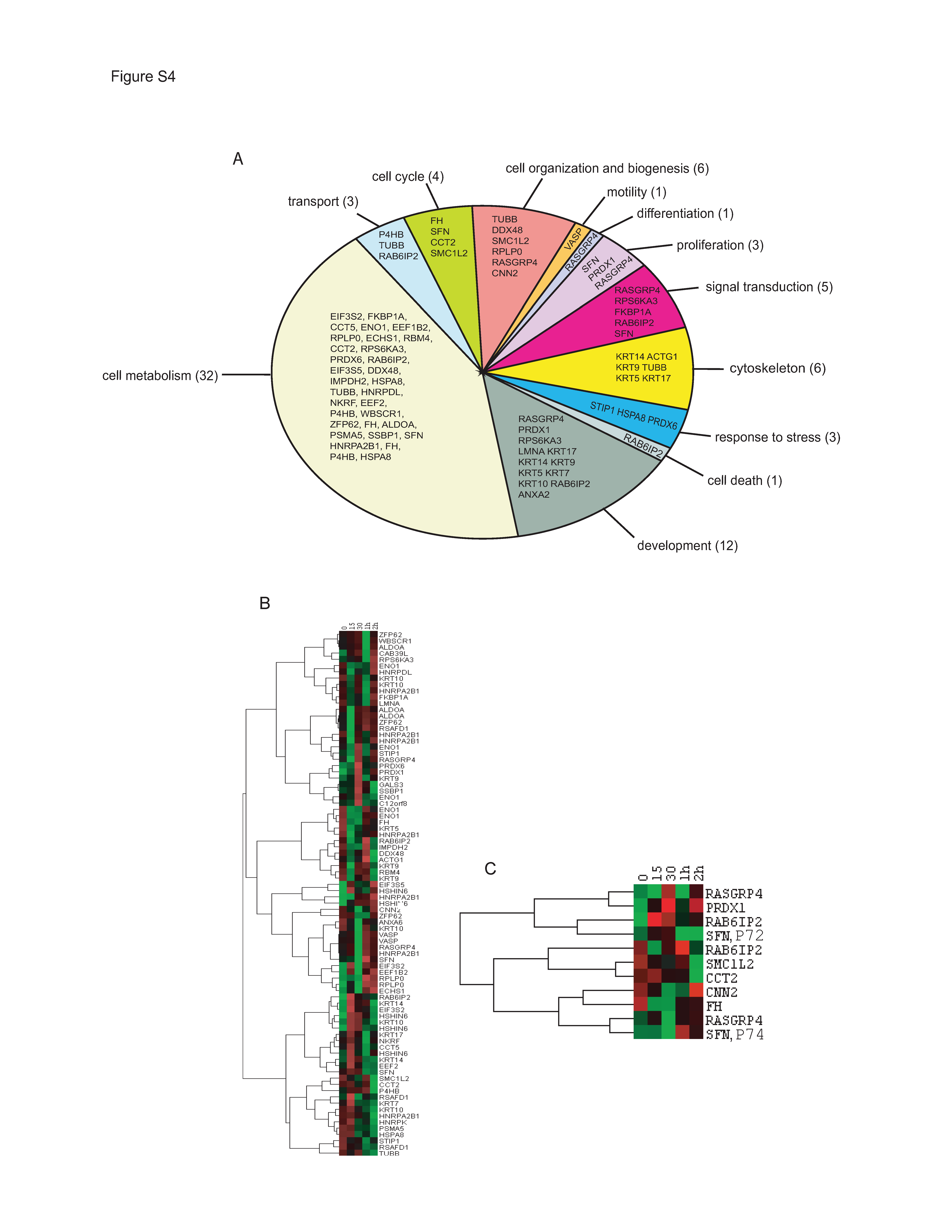

Supplement: Figure S4 — Functional and dynamic clustering of TGFβ1-regulated phosphoproteins. (A) Functional clusters and number of proteins assigned to the clusters are indicated. (B) Heatmap of the TGFβ1-regulated phosphoproteins clustered according to the changes in their expression. (C) Dynamics of phosphoproteins involved in regulation of cell proliferation and cell death. Proteins are annotated in Gene Ontology (GO) terms. (TIF) [file pone.0065163.s004.tif]

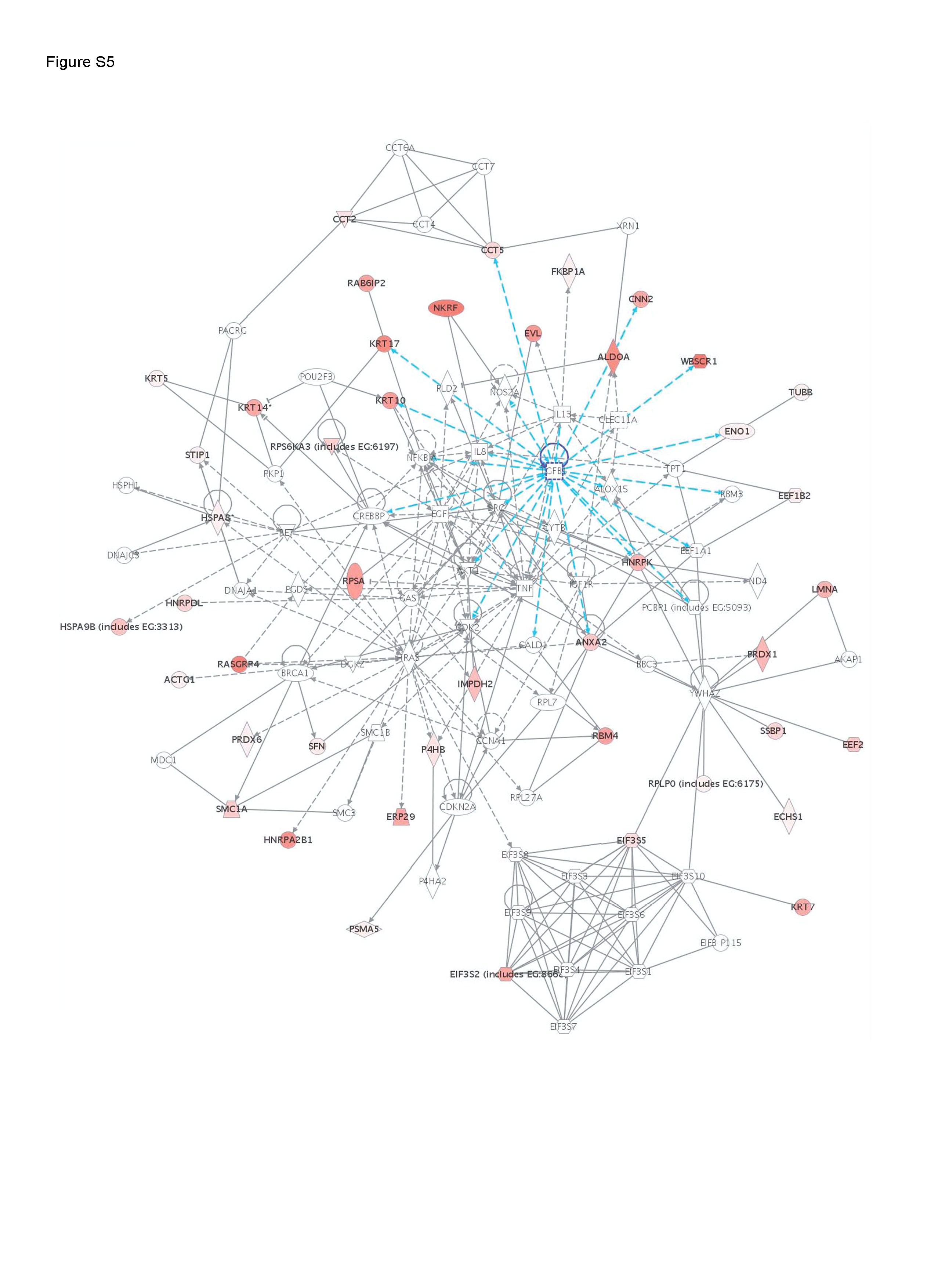

Supplement: Figure S5 — Network of TGFβ1-regulated phosphoproteins. TGFβ1-regulated proteins are presented in a network with their known targets and regulators. Strings between proteins/species represent dependencies which describe physical and/or functional interactions between these species. (TIF) [file pone.0065163.s005.tif]

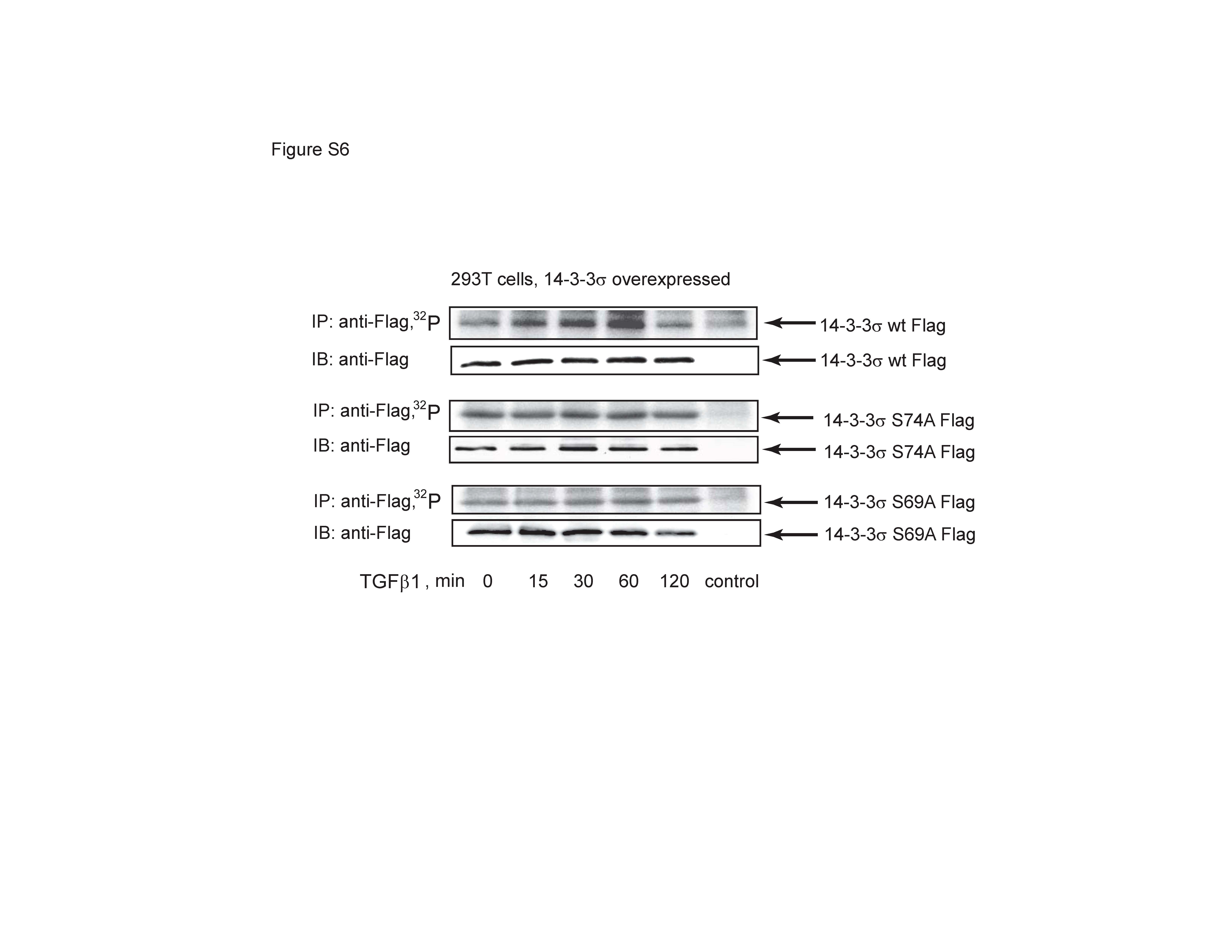

Supplement: Figure S6 — (TIF) [file pone.0065163.s006.tif]

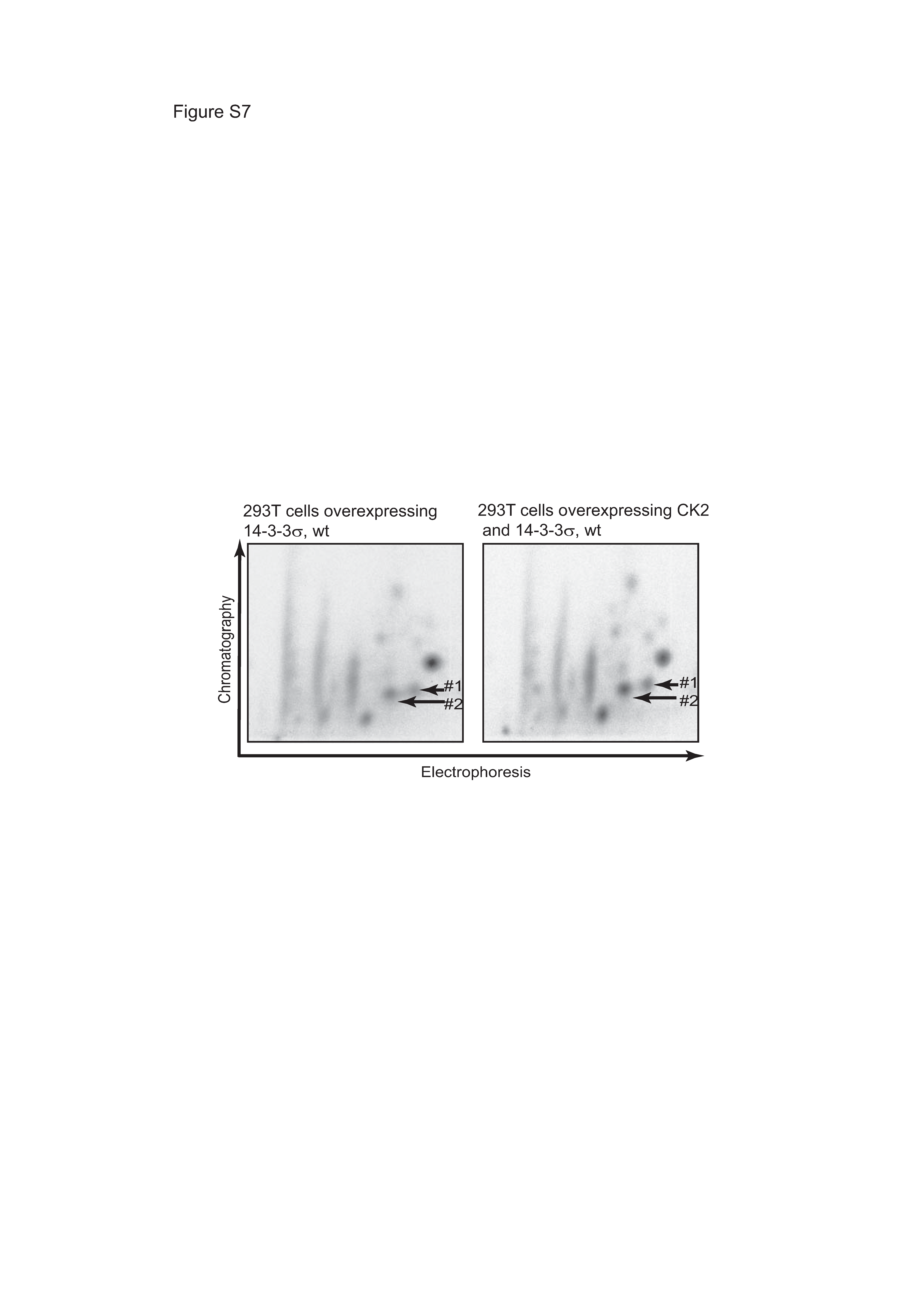

Supplement: Figure S7 — Two-dimensional phosphopeptides mapping showed increase of the phosphorylation of two 14-3-3σ peptides in response to CK2 overexpression. 293T cells were transfected with CK2 expressing construct or control vector and subjected to the two-dimensional phosphopeptides mapping analysis. Migration positions of these phosphopeptides are shown by arrows, as #1 and #2 respectively. (TIF) [file pone.0065163.s007.tif]
